# Characterisation of *Plasmodium falciparum* populations selected on the human endothelial receptors P-selectin, E-selectin, CD9 and CD151

**DOI:** 10.1038/s41598-017-04241-3

**Published:** 2017-06-22

**Authors:** Nahla Galal Metwally, Ann-Kathrin Tilly, Pedro Lubiana, Lisa K. Roth, Michael Dörpinghaus, Stephan Lorenzen, Kathrin Schuldt, Susanne Witt, Anna Bachmann, Henning Tidow, Thomas Gutsmann, Thorsten Burmester, Thomas Roeder, Egbert Tannich, Iris Bruchhaus

**Affiliations:** 1 Bernhard Nocht Institute for Tropical Medicine, Hamburg, Germany; 2 Medical Parasitology Department, Faculty of Medicine-Suez Canal University, Ismailia, Egypt; 3 Department of Chemistry, Institute for Biochemistry and Molecular Biology, University of Hamburg, Hamburg, Germany; 4 Division of Biophysics, Research Center Borstel, Leibniz-Center for Medicine and Biosciences, Borstel, Germany; 5 Institute of Zoology, Biocenter Grindel, University of Hamburg, Hamburg, Germany; 6 Zoological Institute, Department of Molecular Physiology, Christian-Albrechts University Kiel, Kiel, Germany

## Abstract

The ability of the parasite *Plasmodium falciparum* to evade the immune system and be sequestered within human small blood vessels is responsible for severe forms of malaria. The sequestration depends on the interaction between human endothelial receptors and *P. falciparum* erythrocyte membrane protein 1 (*Pf*EMP1) exposed on the surface of the infected erythrocytes (IEs). In this study, the transcriptomes of parasite populations enriched for parasites that bind to human P-selectin, E-selectin, CD9 and CD151 receptors were analysed. IT4_var02 and IT4_var07 were specifically expressed in IT4 parasite populations enriched for P-selectin-binding parasites; eight *var* genes (IT4_var02/07/09/13/17/41/44/64) were specifically expressed in isolate populations enriched for CD9-binding parasites. Interestingly, IT4 parasite populations enriched for E-selectin- and CD151-binding parasites showed identical expression profiles to those of a parasite population exposed to wild-type CHO-745 cells. The same phenomenon was observed for the 3D7 isolate population enriched for binding to P-selectin, E-selectin, CD9 and CD151. This implies that the corresponding ligands for these receptors have either weak binding capacity or do not exist on the IE surface. Conclusively, this work expanded our understanding of *P. falciparum* adhesive interactions, through the identification of *var* transcripts that are enriched within the selected parasite populations.

## Introduction

*Plasmodium falciparum* is responsible for most of the morbidity and mortality accompanying malaria in humans. According to the World Health Organisation, 212 million cases were reported globally in 2015 with an estimated 429,000 deaths^1^.

The ability *of P. falciparum*-infected erythrocytes (IEs) to evade the immune system and be sequestered in small blood vessels of vital organs constitutes the major virulence attribute^2, 3^. IE sequestration underpins the most severe pathological phenotypes observed in malaria, including blood flow obstruction, hypoxia, induction of inflammatory immune responses, endothelial dysfunction, tissue damage and, ultimately, organ failure^2, 4–6^.

The success of *P. falciparum* as a parasite of humans is attributed to the presence of variant surface antigens (VSAs) on the IE surface. These antigens are encoded by five large multi-copy gene families, namely, the *var*, *rif* (repetitive interspersed family), *stevor* (subtelomeric variable open read frame), *surf* (surface-associated interspersed), and *Pfmc-2tm* (*P. falciparum* Maurer’s cleft 2 transmembrane) multi-copy gene families. Their gene products are *P. falciparum* erythrocyte membrane protein 1 (*Pf*EMP1), RIFIN, STEVOR, SURFIN and *Pf*MC-2TM proteins, respectively^7–12^.

*Pf*EMP1 proteins play a major role in cytoadhesion and antigenic variation of IEs. The *Pf*EMP1-encoding *var* genes vary greatly from parasite to parasite, giving rise to an enormous repertoire of *var* genes in nature^8, 13–16^. The expression of *var* genes is mutually exclusive in ring-stage parasites, so that only one *Pf*EMP1 variant is localised on the IE surface at any given time^17^. *Pf*EMP1 proteins consist of a single intracellular domain, a transmembrane domain, and several extracellular Duffy binding-like (DBL) domains (α, β, γ, δ, ζ, and ε) and cysteine-rich interdomain regions (CIDR) (α, β and γ)^18–20^. *Pf*EMP1 proteins can be classified into groups A, B, B/A, C, B/C or E, depending on the chromosomal localisation of the encoding gene, the upstream sequences and *Pf*EMP1 domain composition^8, 21–23^. In addition, an analysis of 399 different *Pf*EMP1 sequences from seven *P. falciparum* genomes allowed the identification of 23 domain cassettes (DCs)^24^.

At least 23 human endothelial receptors or structures interact with IEs^3, 25–30^. Nevertheless, interactions with only a few human endothelial receptors, particularly CD36, intercellular adhesion molecule-1 (ICAM-1) and endothelial protein C receptor (EPCR), have been studied in detail.

Over 80% of *Pf*EMP1 proteins of the *P. falciparum* isolates 3D7 and IT4 contain CIDRα2–6 domains, which are involved in CD36 binding^31^. EPCR was identified as the endothelial receptor of DC8-*Pf*EMP1 and DC13-*Pf*EMP1 (CIDRα1 domain)^26, 32^. In fact, the binding of CIDRα1 to EPCR is associated with severe malaria^26, 33^, and recent studies indicated that the main *Plasmodium var* transcript in severe paediatric malaria patients encodes protein domains predicted to bind EPCR^34, 35^. ICAM-1 binding is linked to the DBLβ3 within DC4-*Pf*EMP1 of group A proteins and to the DBLβ5 domains of group B and C proteins^20, 36–39^. In addition, DC5-*Pf*EMP1 was found to be responsible for the binding to PECAM-1^29^.

IEs also bind to P- and E-selectins^40–44^ and *Pf*EMP1 was suggested to be the interacting partner^45^; however, other studies with various *P. falciparum* isolates did not reproduce E-selectin binding^42, 46^. Recently, two tetraspanins, CD9 and CD151, were shown to interact with IEs^25^, but the identities of their binding partners are unknown.

The current study aimed to identify the as yet unknown ligands of *P. falciparum* that interact with the human receptors P-selectin, E-selectin, CD9 and CD151. Specifically expressed *var* genes were identified within parasite populations enriched for CD9 and P-selectin binding. However, the expression profiles of populations enriched for parasites binding to E-selectin and CD151 were identical to the expression profiles of parasite population exposed to wild-type CHO-745 cells.

## Results

### Endothelial receptor-binding capacity of *P. falciparum* isolates IT4 and 3D7

To identify the putative *Pf*EMP1 ligands responsible for IE binding to various human receptors, the receptors were expressed on the surface of CHO-745 cells, and transcriptomes of ring-stage parasite populations enriched for parasites binding to these receptors were analysed.

**Figure 1:**
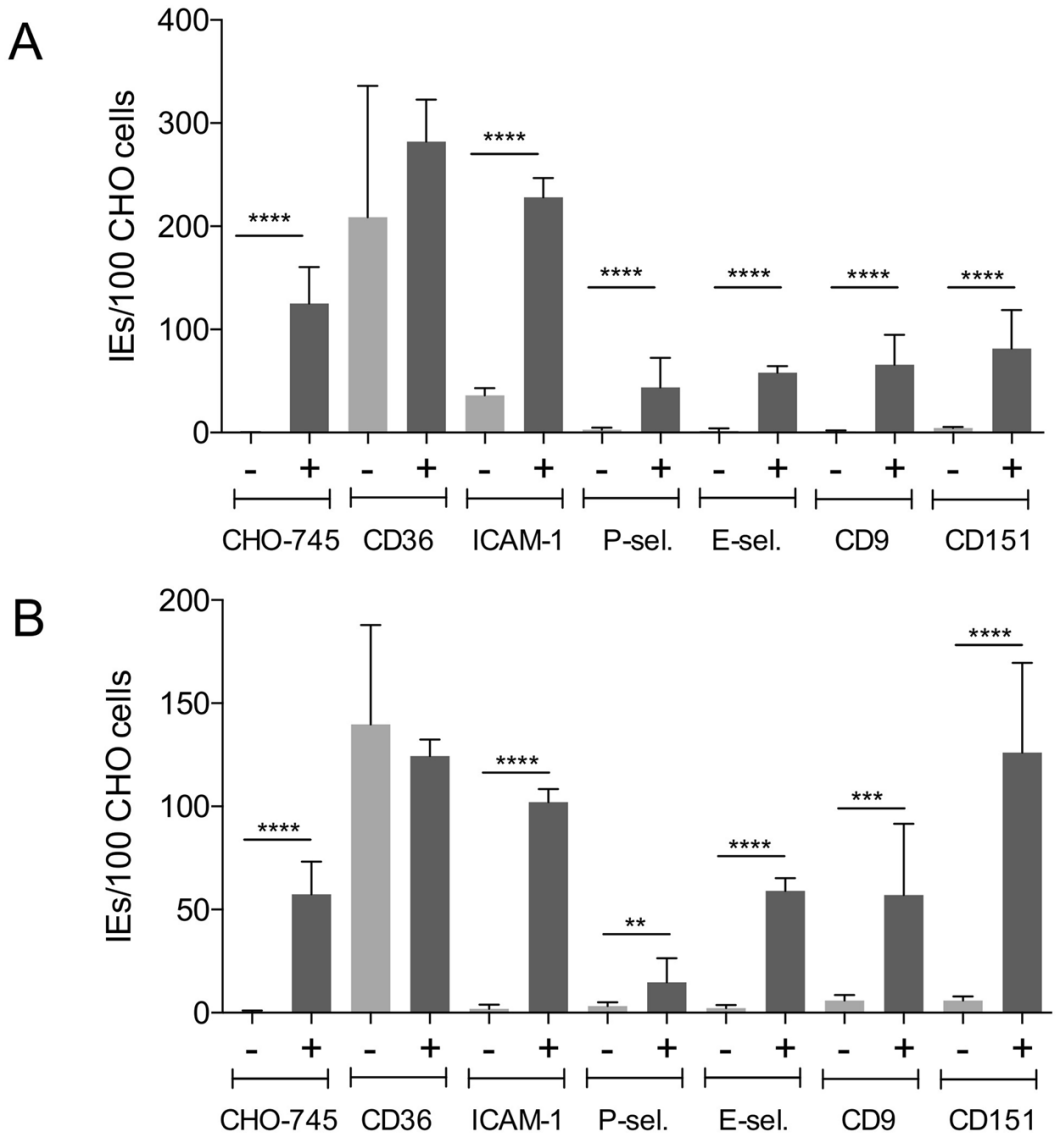
The binding capacity of *P. falciparum* isolates IT4 and 3D7 to wild type CHO-745 cells and to various endothelial receptors before (−) and after (+) enrichment for binding to a receptor of interest. CHO-745 cells expressing the receptor of interest on their surface were used to analyse the binding capacity of isolates IT4 (**A**) and 3D7 (**B**). After incubation of trophozoite-stage IEs with the respective CHO-745 cell monolayer seeded on a coverslip, the slides were washed, fixed with 1% glutaraldehyde and finally stained with Giemsa stain. Bars represent the mean number ± SD of IEs that bound per 100 CHO-745 cells, as determined by light microscopic evaluation. CHO-745, non-transfected CHO-745 WT cells. Statistical significance was determined using the unpaired *t*-test; **p < 0.01, ***p < 0.001, ****p < 0.0001.

In the first stage of analyses, the basic binding capacity of two laboratory strains, IT4 and 3D7, to the receptors of interest was evaluated. Static cytoadhesion assays were performed using trophozoite-stage IEs and transgenic CHO-745 cells with endothelial receptors expressed on their surface as green fluorescent protein (GFP) fusions (Fig. 1, Supplementary Fig. S1A–S1D and Fig. S2). Even after long-term cultivation, large number of *P. falciparum* IT4 parasites could bind to the endothelial cell receptor CD36 even without enrichment (Fig. 1A, Supplementary Fig. S1A) and, on average, 209 bound IEs per 100 CHO-745 cells were detected. Approximately 36 IEs were bound per 100 CHO745 cells expressing ICAM-1 (Fig. 1A and Supplementary Fig. S1B). By comparison, the binding capacity for the other studied receptors and for CHO-745 WT cells was very low. In the case of these receptors, no more than 5 IEs were bound per 100 receptor-expressing CHO-745 cells (Fig. 1A and Supplementary Fig. S1C, S1D). The CD36-binding capacity of *P. falciparum* 3D7 parasites after long-term culture without enrichment was lower than that of the IT4 isolate (Fig. 1B); on average, 140 bound IEs per 100 CHO-745 cells were observed. For all the other receptors studied, including ICAM-1, corresponding to no more than 6 IEs per 100 CHO-745 cells, was observed.

Because of the low proportion of IEs that were able to bind to ICAM-1, P-selectin, E-selectin, CD9 and CD151, parasite populations were then enriched for parasites with a higher capacity to bind to these receptors through repeated rounds of panning assays.

In parallel, we performed enrichment of parasite binding to CHO-745 WT cells. After the first round of selection, an interesting observation was the accumulation of IEs on large, morphologically abnormal CHO-745 cells. These so called “senescent” cells are always present in very low amounts in any normal culture^47, 48^. In subsequent rounds of enrichment, the number of CHO-745-attached IEs increased dramatically (Supplementary Fig. S3A–S3D).

The binding capacity of the various enriched parasite populations was then investigated. As shown in Fig. 1A and B, high numbers of IEs infected by isolate IT4 or 3D7 bound CD36-expressing CHO-745 cells and a further increase of binding was not possible by continued enrichment. For all other analysed receptors and for CHO-745 WT cells, the number of bound IEs increased significantly (44–228 IT4-IEs/100 CHO-745 cells, and 15–140 3D7-IEs/100 CHO-745 cells).

### *var* gene expression profiles in enriched IT4 parasite populations

To identify *var* transcripts produced specifically in enriched parasite populations, three IE populations were generated. The first population comprised parasites after long-term culturing that were representative of the overall parasite population. The second population contained IEs enriched for those that had the ability to bind to CHO-745 WT cells. This allowed the identification of differences between IEs that were able to bind specifically to a receptor of interest and those that bound unknown surface proteins/structures on CHO-745 WT cells. The third population comprised IEs that were enriched on the receptors of interest (Supplementary Fig. S4A–S4C). Using NGS (next generation sequencing), the transcriptome profiles of the parasite populations were determined and evaluated using bioinformatics tools, focusing on *var* gene expression in the early ring-stage parasites (Supplementary Fig. S4D).

**Figure 2:**
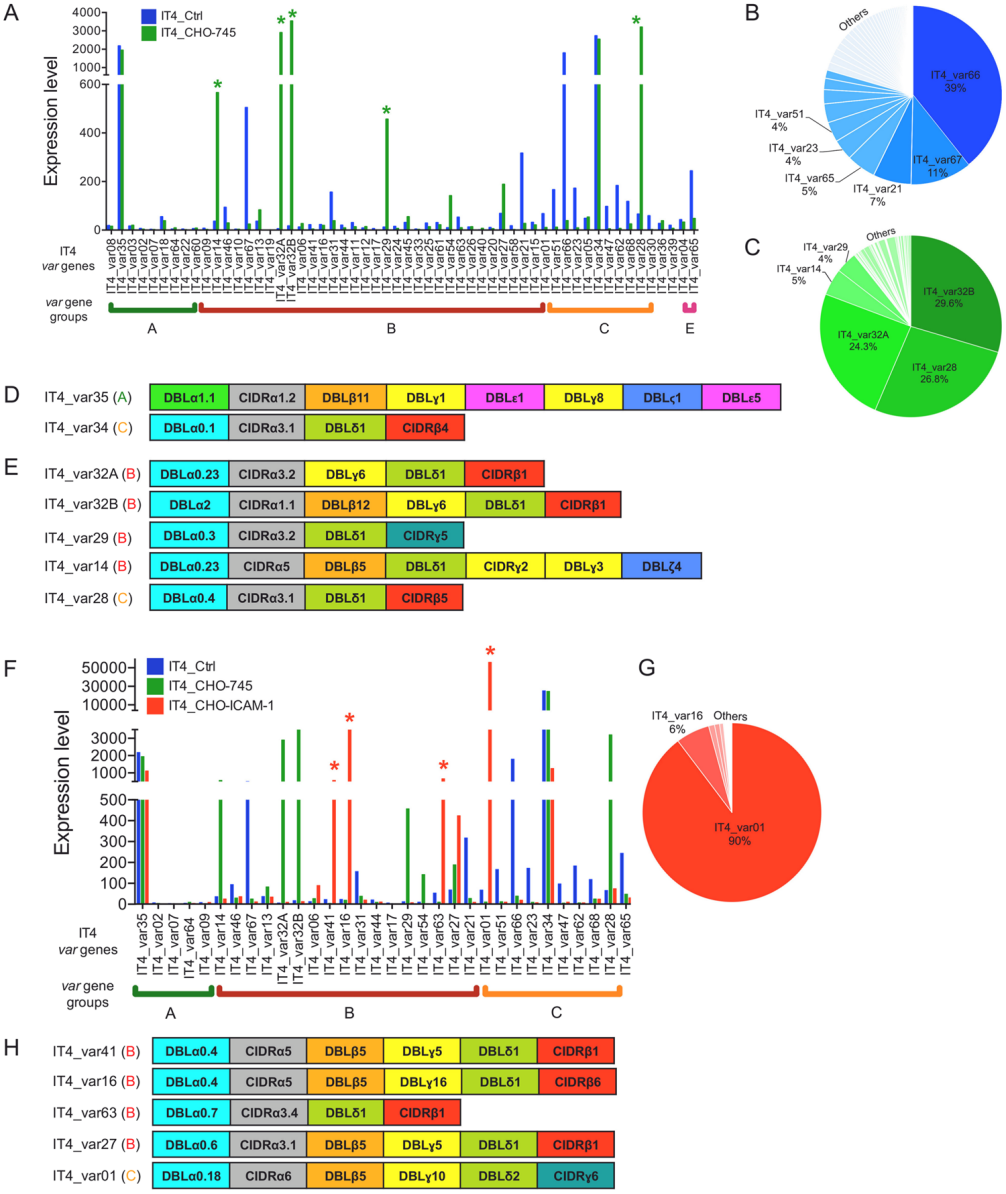
Enrichment of a *P. falciparum* IT4 population for parasites binding to CHO-745 WT cells (IT4_CHO-745) and for ICAM-1–binding parasites (IT4_CHO-745–ICAM-1). (**A**) The expression of a selected set of *var* genes in long-term cultured IT4 parasites (IT4_Crtl) and IT4_CHO-745 (expression level is defined as average of the normalized read counts). *var* genes with a significantly higher expression in IT4_CHO-745 than in IT4_Ctrl are marked by green stars (padj < 0.05). (**B**) Distribution of *var* gene expression in the IT4_Crtl population. (**C**) Distribution of *var* gene expression in the IT4_CHO-745 population. IT4_var34 and IT4_var35 were excluded from the analyses. (**D**) Schematic representation of *Pf*EMP1 proteins encoded by IT4_var35 and IT4_var34 genes with high expression levels in all the investigated parasite populations. (**E**) Schematic representation of *Pf*EMP1 proteins encoded by *var* genes whose expression was significantly increased in IT4_CHO-745 parasites. (**F**) The expression of a selected set of *var* genes in IT4_CHO-745–ICAM-1 parasites compared with gene expression in long-term cultured IT4 parasites (IT4_Crtl) and an IT4 population enriched for parasites binding to CHO-745 cells (IT4_CHO-745). *var* genes with significantly increased expression in IT4_CHO-745–ICAM-1 parasites are marked by red stars (padj < 0.05). (**G**) Distribution of *var* gene expression in the IT4_CHO-745–ICAM-1 population. IT4_var34 and IT4_var35 were excluded from the analyses. (**H**) Schematic representation of *Pf*EMP1 proteins encoded by IT4_var41, IT4_var16, IT4_var63, IT4_var01 and IT4_var27 genes whose expression was increased in IT4_CHO-745–ICAM-1 in comparison with IT4_Crtl and IT4_CHO-745 parasites.

The analysis of the transcriptome of the IT4 isolate after long-term culturing (IT4_Crtl) revealed that nearly all *var* genes were expressed (Fig. 2A and Supplementary Table S1). Most IT4 parasites expressed group A IT4_var35 and group C IT4_var34 genes (Fig. 2A and D). These two genes were also highly expressed in all other IT4 populations analysed (Figs 2A/F, 3A/D and 4A/B, S6A) and were therefore excluded from subsequent analyses. The other highly expressed *var* genes in the IT4_Ctrl population were IT4_var66 (representing 39% of all *var* transcripts), followed by IT4_var67 (11%), IT4_var21 (7%) and IT4_var65 (5%) (Fig. 2A and B).

**Figure 3:**
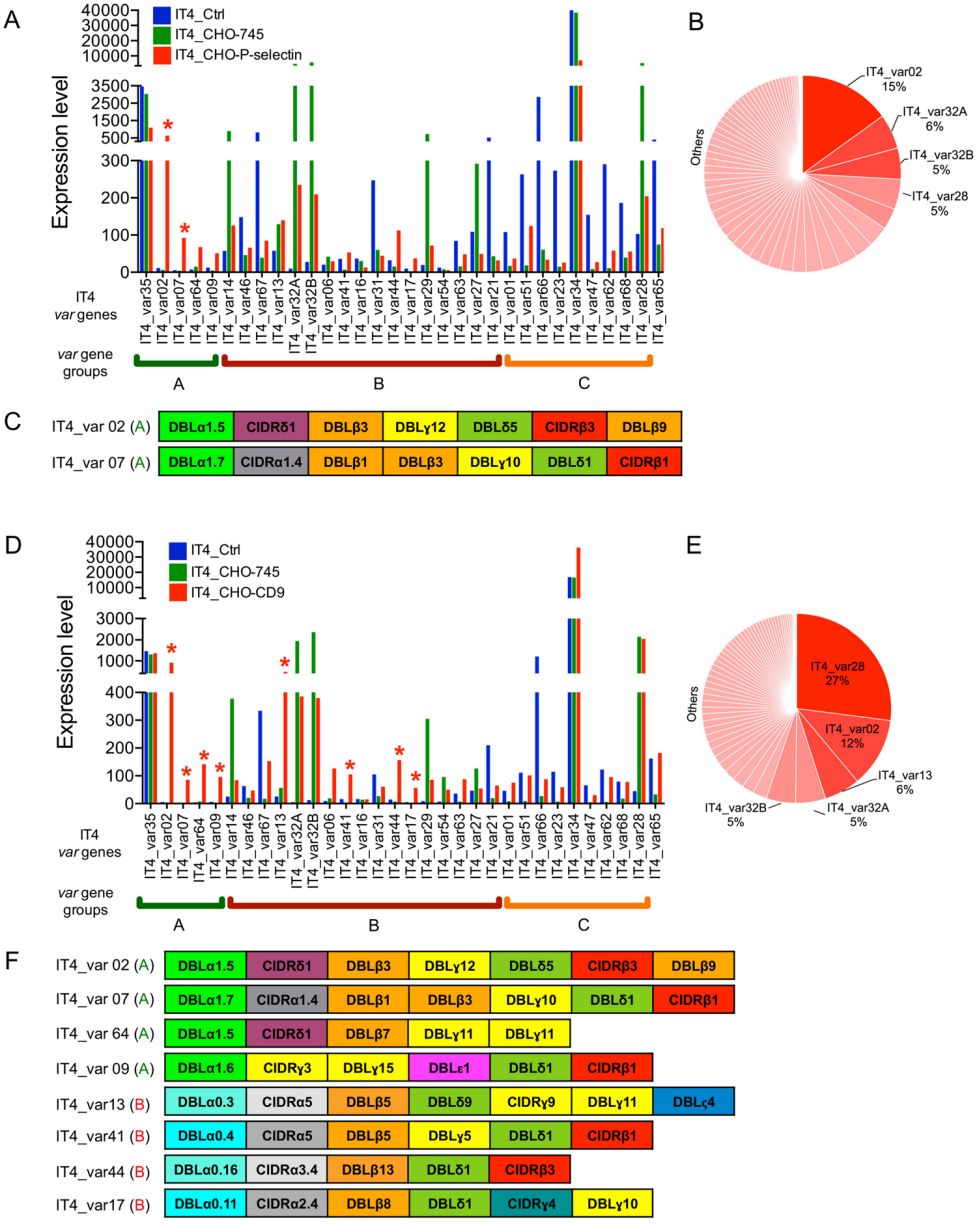
Enrichment of a *P. falciparum* IT4 population for P-selectin–binding parasites (IT4_CHO-745–P-selectin) and for CD9-binding parasites (IT4_CHO-745–CD9). (**A**) The expression of a selected set of *var* genes in IT4_CHO-745–P-selectin parasites in comparison with long-term cultured IT4 parasites (IT4_Crtl) and an IT4 population enriched for parasites binding to CHO-745 cells (IT4_CHO-745) (expression level is defined as average of the normalized read counts). *var* genes with significantly increased expression in IT4_CHO-745–P-selectin parasites are marked by red stars (padj < 0.05). (**B**) Distribution of *var* gene expression in the IT4_CHO-745–P-selectin population. IT4_var34 and IT4_var35 genes were excluded from the analyses. (**C**) Schematic representation of *Pf*EMP1 proteins encoded by IT4_var02 and IT4_var07 genes with a significantly higher expression in IT4_CHO-745–P-selectin than in IT4_Crtl and IT4_CHO-745 parasites. (**D**) The expression of a selected set of *var* genes in IT4_CHO-745–CD9 parasites in comparison with long-term cultured IT4 parasites (IT4_Crtl) and an IT4 population enriched for parasites binding to CHO-745 cells (IT4_CHO-745). *var* genes with significantly increased expression in IT4_CHO-745–CD9 parasites are marked by red stars (padj < 0.05). (**E**) Distribution of *var* gene expression in the IT4_CHO-745–CD9 population. IT4_var34 and IT4_var35 were excluded from the analyses. (**F**) Schematic representation of *Pf*EMP1 proteins encoded by IT4_var02, IT4_var07, IT4_var64, IT4_var 09, IT4_var13, IT4_var41, IT4_var44 and IT4_var17 genes with a significantly higher expression in IT4_CHO-745–CD9 than in IT4_Crtl and IT4_CHO-745 parasites.

**Figure 4:**
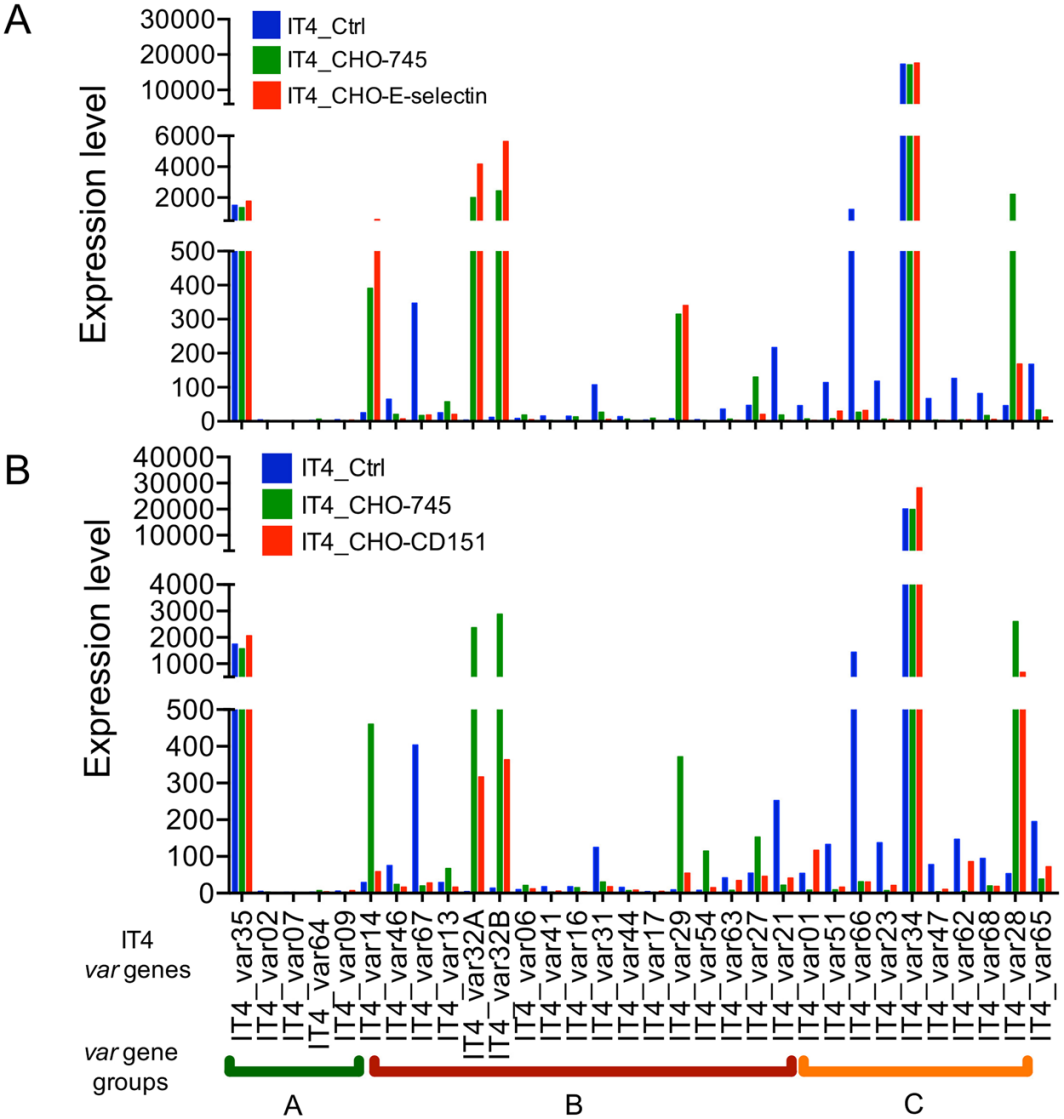
Enrichment of a *P. falciparum* IT4 population for E-selectin– and CD151-binding parasites (IT4_CHO-E-selectin and IT4_CHO-CD151, respectively). The expression of a selected set of *var* genes in IT4_CHO-745–E-selectin (**A**) and IT4_CHO-745–CD151 parasites (**B**) compared with long-term cultured IT4 parasites (IT4_Crtl) and an IT4 population enriched for parasites binding to CHO-745 cells (IT4_CHO-745) (expression level is defined as average of the normalized read counts).

After six to seven rounds of enrichment, an IT4 parasite population with a strong binding capacity to CHO-745 WT cells was selected (Supplementary Fig. S3A–S3D). The transcriptome profile of this population showed a significant enrichment of parasite populations that expressed group B IT4_var14, IT4_var32A, IT4_var32B and IT4_var29 genes, and the group C IT4_var28 gene (representing 5%, 24%, 30%, 4% and 27%, respectively, of all *var* transcripts in the transcriptome) (Fig. 2A,C–E).

As a proof of concept, the transcriptomes of populations enriched for parasites with the ability to bind to ICAM-1 and CD36 were analysed. In the IT4 population enriched for ICAM-1–binding parasites, significant expression of the group B *var* genes IT4_var41, IT4_var16 and IT4_var63, and group C *var* gene IT4_var01, was observed (Supplementary Fig. S5A and Table S1). IT4_var01 was the dominant transcript (representing 90% of all *var* transcripts). In addition, there was a tendency to express group B IT4_var27 gene (Fig. 2F and G). Except for IT4_var63, all the detected *var* genes encode a DBLβ5 domain known to mediate binding to ICAM-1^36, 37^ (Fig. 2H).

The IT4 population enriched for CD36-binding parasites displayed a mixed *var* transcript profile in which IT4_var01, which encodes a protein containing the CD36-binding domain, represented 22% of all *var* transcripts (Supplementary Figs S5B, S6A–C and Table S1). The fact that ca. 80% of all *var* genes encode proteins with the CD36-binding domain^8^ hinders the dominance of a specific *var* gene in the transcriptome profile.

Enrichment of the IT4 populations for P-selectin–binding parasites (Supplementary Fig. S5C and Table S1) resulted in populations where the expression of group A IT4_var02 and IT4_var07 genes was significantly higher (adjusted *p*-value (padj) < 0.05) than in the other two populations (IT4_Ctrl and IT4_CHO-745) (Fig. 3A). IT4_var02 was the dominant *var* transcript (15% of all *var* transcripts). This was followed by IT4_var32A, IT4_var32B and IT4_var28 transcripts (6%, 5% and 5%, respectively) (Fig. 3B), those were also dominant transcripts within IT4_CHO-745 population, suggesting that they were not specific to the P-selectin–binding parasites’ population. Both IT4_var02 and IT4_var07 are group A *var* genes; the head structure of the encoded *Pf*EMP1 proteins lacks the CD36-binding domain^8^. Instead, the IT4_var02 *Pf*EMP1 variant contains two DCs, DC16 (DBLα1.5–CIDRδ1) and DC5 (DBLγ12–DBLδ5–CIDRβ3–DBLβ9), and the IT4_var07 *Pf*EMP1 variant contains DC13 (DBLα1.7–CIDRα1.4) (Fig. 3C).

Compared with the IT4_Ctrl and IT4_CHO-745 populations, eight *var* genes were differentially expressed in the IT4 population enriched for CD9-binding parasites (padj < 0.0001) (Fig. 3D, Supplementary Fig. S5D and Table S1). Four of these (IT4_var02, IT4_var07, IT4_var64 and IT4_var09) were group A *var* genes, and the other four (IT4_var13, IT4_var41, IT4_var 44 and IT4_var17) were group B *var* genes. The dominant *var* transcript was IT4_var28 (27%), followed by IT4_var02 (12%), IT4_var13 (6%), IT4_var32A (5%) and IT4_var32B (5%) (Fig. 3B). Three of the dominant *var* genes (IT4_var28, IT4_var32A and IT4_var32B) were also highly expressed in IT4_CHO-745 parasites, and were therefore suggested not to the specific for the IT4_CD9 population. The domain structure of proteins encoded by the four differentially expressed group A *var* genes comprises a head structure with the unique DBLα1 domains, and CIDRδ1 (in IT4_var02 and IT4_var64), CIDRα1.4 (in IT4_var07) or DBLγ3 (in IT4_var09). The four group B variants contain the classical head structure responsible for CD36 binding; in addition, IT4_var13- and IT4_var41-encoded proteins have an ICAM-1−binding domain (Fig. 3F).

The enrichment for the ability to bind E-selectin and CD151 resulted in an IT4 population that highly expressed IT4_var14, IT4_var32A, IT4_var32B and IT4_var28 genes. High similarity with the *var* gene expression profile of the IT4_CHO-745 population was observed (Fig. 4A, B). These results might suggest that both IE populations (IT4_CHO–E-selectin and IT4_CHO–CD151) were selected based on binding to an unknown receptor/structure on the CHO-745 WT cell surface, rather than based on their ability to bind E-selectin or CD151, respectively (Supplementary Table S1).

### *var* gene expression profiles in enriched 3D7 parasite populations

**Figure 5:**
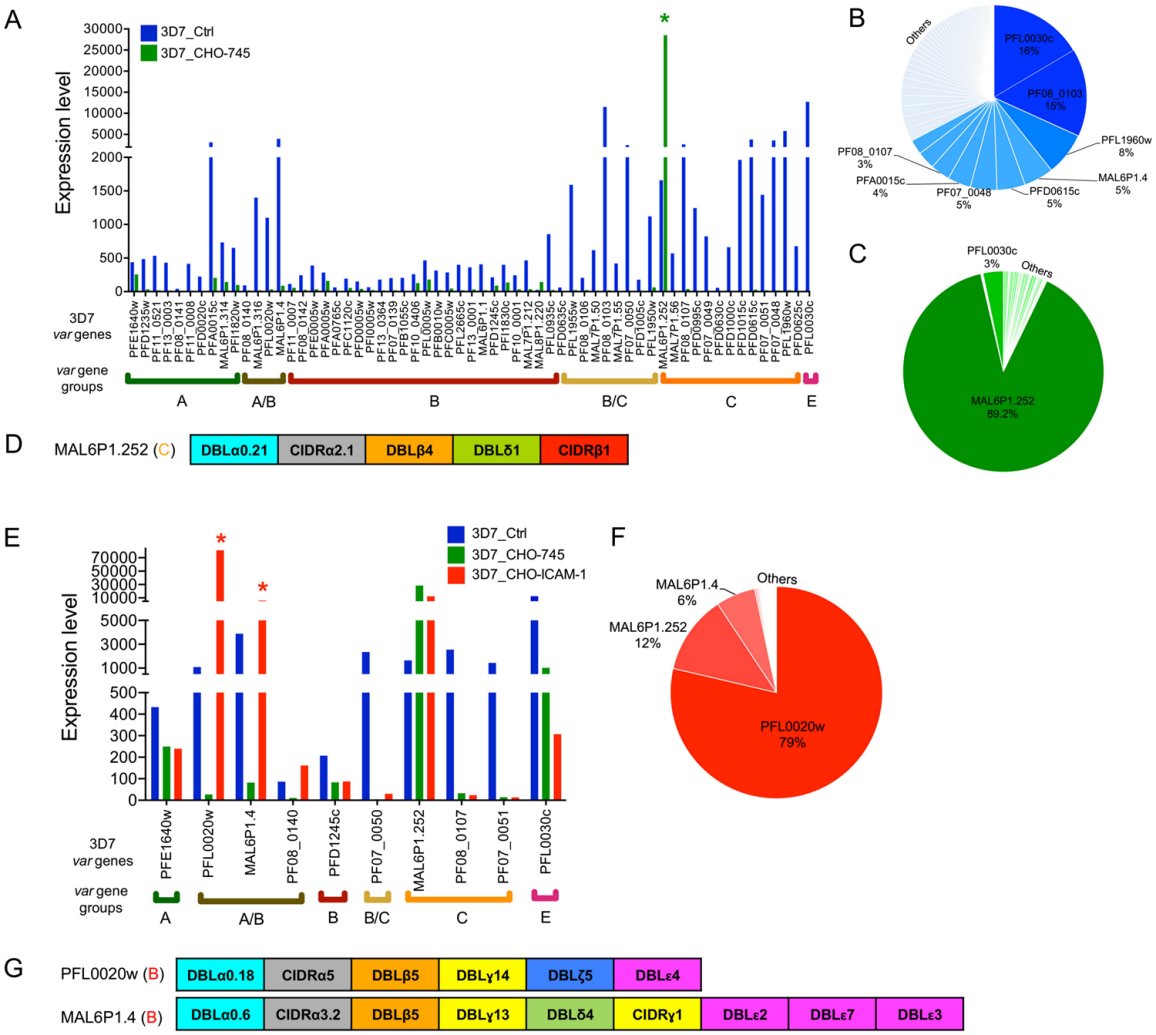
Enrichment of a *P. falciparum* 3D7 population for parasites binding to CHO-745 WT cells (3D7_CHO-745) and for ICAM-1–binding parasites (3D7_CHO-ICAM-1). (**A**) *var* gene expression in long-term cultured 3D7 parasites (3D7_Crtl) and 3D7_CHO-745 parasites (expression level is defined as average of the normalized read counts). A *var* gene with significantly increased expression in 3D7_CHO-745 parasites in comparison with 3D7_Ctrl parasites is marked by a green star (padj < 0.05). (**B**) Distribution of *var* gene expression in the 3D7_Ctrl population. (**C**) Distribution of *var* gene expression in the 3D7_CHO-745 population. (**D**) Schematic representation of *Pf*EMP1 protein encoded by the MAL6P1.252 gene with significantly increased expression in 3D7_CHO-745 parasites. (**E**) The expression of a selected set of *var* genes in 3D7_CHO-745–ICAM-1 parasites in comparison with long-term cultured 3D7 parasites (3D7_Crtl) and a 3D7 population enriched for parasites binding to CHO-745 cells (3D7_CHO-745). *var* genes with significantly increased expression in the 3D7_CHO-745–ICAM-1 parasites are marked by red stars (padj < 0.05). (**F**) Distribution of *var* gene expression in the 3D7_CHO-745–ICAM-1 population. (**G**) Schematic representation of *Pf*EMP1 proteins encoded by PFL0020w and MAL6P1.4 genes with significantly higher expression in 3D7_CHO-745–ICAM-1 than in 3D7_Crtl and 3D7_CHO parasites.

The long-term 3D7 isolate culture (3D7_Crtl) analysed in this study had a stable *var* gene expression profile, with nearly all *var* genes expressed (Fig. 5A). The PFL0030c *var* gene dominated with 16%, followed by PF08_0103, PFL1960w and MAL6P1.4 (15%, 8% and 5%, respectively) (Fig. 5B). Transcriptome analyses of the 3D7 population enriched for CHO-745 cell-binding parasites (3D7_CHO-745) revealed differential expression of MAL6P1.252 (padj ≤ 0.05) in comparison with the 3D7_Ctrl population (Supplementary Table S2). MAL6P1.252 was the dominant *var* transcript (89% of all *var* transcripts), followed by PFL0030c (only 3%) (Fig. 5A and C). The predicted protein encoded by MAL6P1.252 contains the classical head structure required for binding to CD36 (Fig. 5D).

Parasite populations enriched for ICAM-1–binding parasites differentially expressed the group B *var* genes PFL0020w (79% of all *var* transcripts) and MAL6P1.4 (6% of all *var* transcripts) in comparison with the two control populations (Fig. 5E and F; Supplementary Table S2). These two *var* genes encode *Pf*EMP1 molecules that contain DBLβ5 domains known to bind to ICAM-1 (Fig. 5G). High expression was also noted for MAL6P1.252. Although MAL6P1.252 contains a CD36-binding domain, an ICAM-1–binding domain is not present (Fig. 5D).

Results of the enrichment of a 3D7 population for CD36-binding parasites were the same as for the IT4 isolate, i.e., no differentially expressed genes were detected in comparisons with the 3D7_Ctrl and 3D7_CHO-745 populations (Supplementary Fig. S7A and Table S2). The 3D7_CD36 population predominantly expressed PF07_0050 (39% of all *var* transcripts), followed by MAL6P1.252 (15%), PF08_0107 (16%) and PF07_0051 (10%) (Supplementary Fig. S7B). These four *var* genes encode *Pf*EMP1 variants that contain CIDRα2–6 domains responsible for CD36 binding (Supplementary Fig. S7C).

**Figure 6:**
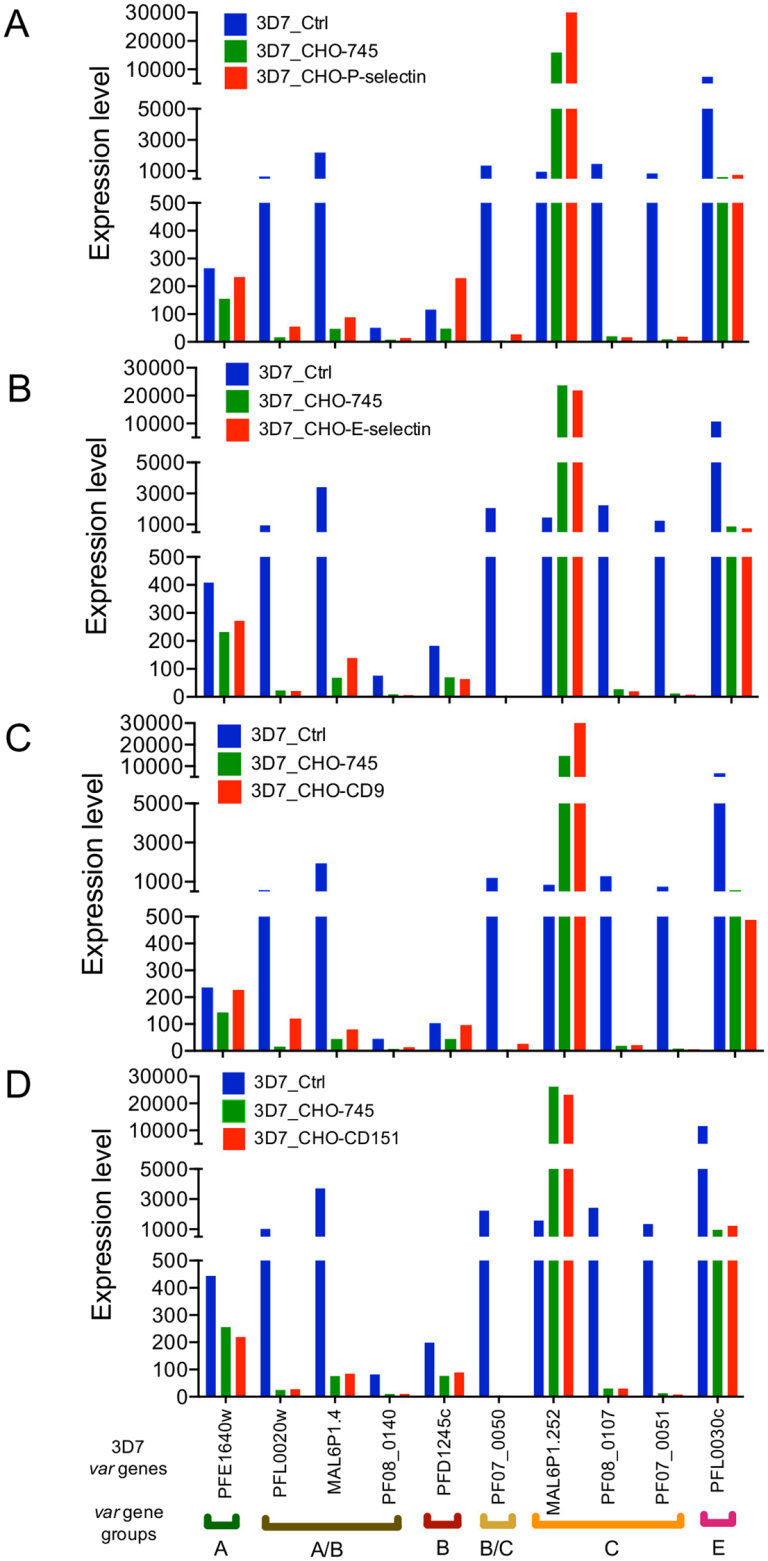
Enrichment of a *P. falciparum* 3D7 population for selectin- and tetraspanin-binding parasites. The expression of a selected set of *var* genes in parasite populations enriched for parasites binding to P-selectin (**A**), E-selectin (**B**), CD9 (**C**) or CD151 (**D**), in comparison with long-term cultured 3D7 parasites (3D7_Crtl) and a 3D7 population enriched for parasites binding to CHO-745 cells (3D7_CHO-745) (expression level is defined as average of the normalized read counts).

Figure 6A–D demonstrates our attempts at enriching the 3D7 population for parasites binding to P-selectin, E-selectin, CD9 and CD151. The expression of all *var* genes was dramatically reduced except for the MAL6P1.252 gene, which was highly expressed in all enriched populations. In all of the enriched populations the expression profiles were comparable with those in the 3D7_CHO-745 population (Supplementary Tables S11–S14).

### Non-*var* multi-copy gene family profiles in enriched parasite populations

Although the current study was designed with a focus on the expression of the *var* genes during the early ring-stage (6–8 h after invasion), it also allowed comparative expression analysis of other multi-copy gene families at this time point. The majority of genes belonging to the *rif*, *stevor, pfmc2tm* or *surf* families were either not expressed or expressed at very low levels in the various parasite populations at the investigated time point.

Considering IT4 isolate (Supplementary Tables S3–S9, S17), three *rif* genes, PFIT_0400300.1, PFIT_0400300.2 and PFIT_bin04400, were approximately equal and relatively high expressed in all parasite populations. Expression of two *rif* genes, PFIT_0616600 and PFIT_bin00500, tended to be higher in the IT4_CHO–ICAM-1 population. In the population enriched for CD36-binding parasites, significant high expression of one *rif* gene (PFIT_0811700) was observed. In addition, the PFIT_0537100, PFIT_1150800 and PFIT_bin04900 *rif* genes tended to be highly expressed. In the transcriptome of IT4_CHO-P-selectin IEs, significant expression of the PFIT_0801600 *rif* gene was observed. PFIT_bin07800 *rif*, PFIT_bin05800 *stevor* and PFIT_1301050 *surf* tended to be highly expressed. The expression of three *surf* genes, PFIT_0803400, PFIT_1301000 and PFIT_0422600, was significantly higher in the IT4_CHO–P-selectin population. In the IT4_CD9 population, four *rif* genes (PFIT_0801600, PFIT_0800200, PFIT_bin03400 and PFIT_bin07800) and four *surf* genes (PFIT_0803400, PFIT_1301000, PFIT_0422600 and PFIT_1301050) were differentially expressed. In addition, the expression of two *stevor* genes, PFIT_0901600 and PFIT_bin05800, was also higher in the IT4_CHO–CD9 population than that in the respective controls (Supplementary Table S17).

Within 3D7 isolate (Supplementary Tables S10–S16, S18), the expression levels of two *rif* genes, PF3D7_0401600.1 and PF3D7_0401600.2, were approximately equal and high in all the parasite populations. Interestingly, most *rif* genes were highly expressed in 3D7_CHO-745 populations. Considering the *stevor* genes, PF3D7_0102100 tended to be highly expressed in the CD9-binding enriched population and PF3D7_0115400 was highly expressed in the ICAM-1–binding enriched population. The expression of only one *surf* gene (PF3D7_1301800) was equally high in all the enriched parasite populations and in the controls. *Pf*mc2tm gene family was expressed at various levels in the control and enriched populations (Supplementary Table S18).

## Discussion

A complete *Pf*EMP1 interactome map is still not available. In the current study, we were aiming to predict putative *P. falciparum var* genes that encode ligands for different endothelial receptors. We established and combined three approaches (cellular, molecular and bioinformatics) that enabled us to perform a comprehensive analysis of different parasite populations enriched for parasites binding to different endothelial receptors exposed on the surface of transgenic CHO-745 cells. The robustness of the methods used in this study was confirmed by analysing the transcriptomes of two different isolate populations (IT4 and 3D7) enriched for ICAM-1– and CD36-binding parasites. The respective parasite populations expressed *var* genes that encode *Pf*EMP1 molecules containing binding domains for ICAM-1 and CD36, respectively (DBLβ5 and CIDRα2–6, respectively). As was also shown by Petter and colleagues, the dominant transcript in parasites selected on ICAM-1 was PFL0020w^49^. In the case of the IT4 isolate, as previously described, all six *Pf*EMP1 molecules (IT4_var01, IT4_var13, IT4_var14, IT4_var16, IT4_var27 and IT4_var41) containing the DBLβ5 domain could bind to ICAM-1^36, 37, 50^. Of these, IT4_var01, IT4_var16, IT4_var27 and IT4_var41 were identified as dominant transcripts in ICAM-1–enriched parasites in this study, with 90% of all *var* transcripts attributed to IT4_*var*01.

In addition to the interaction of IEs with the CD36 and ICAM-1 receptors, it was also reported that IEs can adhere to other molecules, including lectins, P-selectin and E-selectin, and tetraspanins, CD9 and CD151^25, 39, 43, 44^. However, in contrast to ICAM-1 and CD36, *P. falciparum* ligands participating in these binding events are as yet unknown.

In the present study, following comparison with the respective controls, IT4_var02 was identified as the dominant transcript in IT4 parasite populations enriched for P-selectin– and CD9-binding parasites. The *Pf*EMP1 variant encoded by this gene contains a head structure with a non-binding CD36 domain forming DC16, followed by DC5. Parasites expressing IT4_var02 exhibit binding affinity for transformed human bone marrow endothelial cells, and this binding ability could not be inhibited by anti-EPCR antibodies^26^. Furthermore, strong binding affinities of DC5 of the 3D7 isolate (PF11_0008 gene) and IT4 isolate (IT4_var02 gene) for transformed bone marrow endothelial cells expressing both ICAM-1 and PECAM-1 were also reported. Inhibition of the binding by anti-ICAM-1 antibodies was not successful; the binding was inhibited by anti–PECAM-1 antibodies^29^.

Moreover, IT4_var07 was differentially expressed in both IT4_CHO-P-selectin and IT4_CHO-CD9 populations. The IT4_var07-encoded *Pf*EMP1 variant contains DC13^8^. High levels of DC13 transcript were reported in patients with severe malaria^33^. IEs expressing DC13 bind to different endothelial cell types, and it has been proposed that DC13 mediates binding to EPCR via the CIDRα1 domain subclass^24, 26^. Binding of parasite populations expressing IT4_var07 to endothelial cell lines in the brain, lung and dermis under shear stress conditions was also demonstrated. Surprisingly, when EPCR was blocked, the binding to bone marrow and lung endothelial cells was inhibited, but the IEs continued to bind to the brain and dermal endothelial cells, confirming the versatility of IE receptor affinities^51^.

In this study, we observed eight highly expressed *var* genes within CD9 enriched populations, which is somehow puzzling. Four of the genes encode for group A *Pf*EMP1 (IT4_*var*02, IT4_*var*64, IT4_*var*09 and IT4_*var*07) and the rest encode for group B *Pf*EMP1 (IT4_*var*44, IT4_*var*13, IT4_*var*17 and IT4_*var*41), rendering some difficulty to predict the exact interacting *Pf*EMP1 domain(s).

The question of whether P-selectin and CD9 play a role in cerebral malaria (CM) requires further investigation. In *Plasmodium berghei*-infected mice, which can be used as a model of experimental CM, mice lacking P-selectin are protected from malaria-induced death^52, 53^. Up-regulation of P-selectin in the endothelial lining of several organs was reported for a murine *P. berghei* infection. However, the importance of P-selectin in CM is controversial; one study showed up-regulation of P-selectin in the brain vessels of malaria-susceptible mice but not in CM-resistant mice, whereas another study reported a significant increase in P-selectin levels in resistant mice in comparison with control mice^52, 53^. Elevated levels of soluble P-selectin in the sera of patients with severe malaria in comparison with patients with non-severe symptoms were also reported^54^.

Recently, CD9 was identified as a new interaction partner of IEs^25^; however, it is not known whether CD9 plays a role in the development of malarial pathogenesis. CD9 plays a role in the leukocyte aggregation cascade, where it mediates leukocyte aggregation in the brain through endothelial adhesive platforms (EAPs), i.e., adhesive clusters containing CD9 and ICAM-1. Upon leukocyte binding via CD9, the EAPs rise above the endothelial surface to facilitate leukocyte capture^55^. CD9 also plays a role in some infectious diseases, like in HIV or bacterial infections^56, 57^. All these data indicate the prime importance of the identification of the parasite’s ligand(s) in the interactions mentioned above.

We were unable to identify a parasite population binding to E-selectin. Until now, neither the role of E-selectin in CM nor its *Pf*EMP1 ligand has been identified. Post-mortem brain examination of patients who died from CM in Thailand revealed up-regulation of E-selectin in the endothelial lining. In the same study, it was also possible to select IEs for binding to E-selectin^40^ In a study from the same region, 60 patient *Plasmodium* isolates from patients were tested for adherence to CD36, ICAM-1, VCAM-1 and E-selectin; the binding to the latter two was negligible^46^. Furthermore, no binding of E-selectin was detected under flow conditions^41^. Recently, Janes and colleagues tested the binding capacity of IEs that expressed group A *var* genes for CHO-745 transfectants with superficial E-selectin expression; only minimal binding was observed^39^. The controversial data mentioned above may support the observations in the current study that suggest that E-selectin plays only a minor or no role in the cytoadhesion of IEs to the vascular endothelium.

Similarly to CD9, CD151 was recently shown to bind IEs^25^. Based on the current analysis of the two laboratory isolates, we conclude that CD151 has only minor relevance for cytoadhesion to IEs because the dominant *var* gene transcripts in populations enriched for CD151-binding parasites were exactly the same as those in the CHO-745 WT-grown populations.

A high and almost identical expression pattern of two *var* genes, IT4_var35 (*var*1, pseudogene) and IT4_var34, was observed in all IT4 populations investigated. IT4_var35 is a truncated gene that lacks the exon 2 sequence and has a shortened acidic terminal segment^8^. It has been postulated that pseudogenes act as a *var* diversity archive, similarly to variant surface glycoproteins in *Trypanosoma* ^58^. We also observed a high and unchanging expression of PFIT_bin09700. BLAST analysis identified this mRNA as a fragment of IT4_var34; it appears that this gene is fragmented (exists as two fragments) in the IT4 isolate used in this study, which might affect its translation to a full-length surface *Pf*EMP1 protein.

We observed an interesting binding phenotype of IEs to wild-type CHO-745 cells. Especially at the first rounds of enrichment large numbers of IEs adhered in a clump-like fashion to morphologically abnormal (rarely occurring) CHO-745 cells. These cells were larger than normal^59^ with many cytoplasmic vacuoles, large or multiple nuclei, an increased number of lysosomes and an extended Golgi network. The cells with such abnormal morphology are known as ‘senescent cells’; they lose their ability to multiply but remain metabolically active^48, 60, 61^. Strong adhesive molecules are present on the surface of senescent endothelial cells^62^. For example, the expression of a gene encoding CD44 is higher in senescent cells than in younger cells. A higher binding capacity of monocytes clusters to CD44 for senescent endothelial cells was also reported^63^.

In another study, the binding of IEs to wild-type CHO-745 cells was also observed. The authors attributed this to the presence of NCAM on the surface of CHO-745 cells because the binding was partially blocked by NCAM-specific antibodies. Interestingly, they also observed clumpy collections of IEs and described them as ‘macro aggregates’ composed of hundreds of IEs^64^. The answer to the question of whether CD44, NCAM, both or additional proteins are responsible for the interaction with IEs remains elusive^65^.

The expression of MAL6P1.252 in the four different 3D7 populations (3D7_CHO–P-selectin/–E-selectin/–CD9/–CD151) was surprising. Frank and colleagues concluded that the *var* genes possess different intrinsic switching rates depending on the *var* gene subtype. They used a clonal parasite population that exclusively expressed the central *var* gene MAL6P1.252. No change in *var* gene expression was observed in this clone over 13 weeks of continuous growth, indicating an immeasurably low off-rate for this locus^66^. Thus, presumably, the populations expressing MAL6P1.252 interact with a receptor on CHO-745 cells. Because of the continuous selection and the low expression of group A *var* genes in the starting culture, these populations dominated and, given their low switching *var* rate, it was impossible to select any other parasite population that could bind P-selectin or CD9.

Recent studies suggest that *Pf*EMP1 is not the main variable surface antigen during a chronic *P. falciparum* infection^67^. The analysis of the expression profiles of other multi-copy gene families revealed an interesting differential expression of *surf* genes in populations enriched for the P-selectin– and CD9-binding parasites. SURFIN was recently described as a polymorphic antigen encoded by 10 *surf* genes, and it was suggested that SURFINs are crucial for parasite survival, but this is still under investigation^12^. The *rif* genes are transcribed 12–27 h post invasion^68^, but, even though the parasite populations were harvested at earlier time points in the current study, we observed a tendency for high expression of some of the *rif* genes in CD36-, ICAM-1–, P-selectin– and CD9-binding populations. This emphasises the many open questions regarding the exact functions of RIFIN proteins in cytoadhesion.

Taken together, the current study predicted *Pf*EMP1 candidates that might interact with P-selectin and CD9. The most interesting finding herein was that the identified *Pf*EMP1 variants belong to group A *Pf*EMP1s, which is a frequent finding in *Plasmodium* isolates from patients with severe malaria. As a proof of concept, ICAM-1 and CD36 ligands were identified. Moreover, we were not able to identify binding partners for E-selectin and CD151. The reason for this may be that the binding to these receptors is weaker, than the binding to the unknown receptor on CHO-745 cells or they do not play a role in cytoadhesion of IEs. Finally, an interesting phenotype concerning the binding of IEs to senescent cells in the CHO-745 cell culture was observed. Further investigations are required to uncover the molecular backgrounds of these interactions, especially the respective *Pf*EMP1 domains responsible for binding.

## Methods

### Parasite culture

*P. falciparum* isolates IT4 (FCR3S1.2) and 3D7 were cultivated with human O+ erythrocytes (5% haematocrit) in the presence of 10% human serum A+ (Interstate blood bank, Hamburg, Germany), according to standard procedures^69^. Parasite cultures were synchronised once a week using 5% sorbitol^70^.

### CHO-745 cell transfection and culture

CHO-745 cells defective in glycosaminoglycan biosynthesis (CHO-745; American Type Culture Collection no. CRL-2242) were used in this study. CD36, ICAM-1, P-selectin and E-selectin cDNAs were cloned into pAcGFP1-N1 (Clontech Laboratories), whereas CD9 and CD151 were cloned into pEGFP-N1 (Clontech Laboratories). CHO-745 cells were transfected using Lipofectamine 2000 (Invitrogen) according to the manufacturer’s protocol. The plasmids were kindly provided by Rolf Horstmann (Bernhard Nocht Institute for Tropical Medicine, Hamburg), and the transfectants were generated as previously described^25, 71^.

Transfected CHO-745 cells were cultivated at 37 °C and under 5% CO_2_ in Ham’s F-12 medium (PAA) supplemented with 10% foetal calf serum (PAA) and penicillin-streptomycin. Neomycin (G418; stock concentration 50 mg/mL; with final concentration, 0.7 mg/mL) was used as a selective agent for the transfected cells.

### Immunofluorescence analyses of endothelial receptors

CHO-745 cells expressing the receptors under investigation were grown on coverslips and fixed with 2% followed by 4% para-formaldehyde. Each CHO-745 cell line was labelled using the respective antibody (αCD62E (1:33, R&D Systems), αCD62P (1:33, Santa Cruz Biotechnology), αCD151 (1:33, Rolf Horstmann, BNITM, Hamburg, Germany), αCD54 (1:50, eBioscience), αCD9 (1:50, Chemicon International), αCD36 (1:50, R&D Systems), which was then conjugated to Alexa-Fluor-594 (Invitrogen) as a secondary antibody. Nuclei were stained with Hoechst-33342 staining (1:1000) (Sigma). The cover slips were then mounted with DAKO fluorescent mounting medium and examined under a fluorescent microscope (Zeiss Axioskop2 plus-Carl Zeiss AG and EVOS FL-Thermofisher). Cells incubated with secondary antibody alone did not show fluorescence in any experiment (Supplementary Fig. S2).

### Static binding assay

The static cytoadhesion assays were performed as previously described^25^. The transfected CHO-745 cells were seeded onto coverslips (13 mm) at a density of 30,000 cells/mL 2 d before the assay. On the day of the assay the phenotype and confluence level of CHO-745 cells was controlled under light microscope. The assay was performed with 50–100% (ideal: 90%) confluent monolayer CHO-745 cells. Highly synchronised trophozoite-stage parasite culture (5% parasitaemia, 1% haematocrit) was suspended in the binding medium (RPMI 1640 medium supplemented with 2% glucose, pH 7.2). The parasite cell suspension was added to CHO-745 cells expressing only GFP (to allow IE pre-absorption) for 1 h at 37 °C and under 5% CO_2_, with orbital shaking every 15 min. Next, the pre-absorbed suspension was incubated with receptor-expressing CHO-745 cells for 1 h at 37 °C and under 5% CO_2_, with gentle shaking in all directions every 15 min. The coverslips were then washed with the binding medium to remove unbound erythrocytes. Cells adhering to the coverslips were fixed with 1% glutaraldehyde in phosphate-buffered saline for 30 min at room temperature. Finally, the cells were stained with a filtered Giemsa/Weisser buffer solution (1:10). The number of adherent IEs was determined by counting 300 CHO-745 cells under a light microscope. Assays were conducted three times in triplicate. The data are expressed as the mean with standard deviation (SD).

### Selection and enrichment for IEs binding to the receptors of interest

Two days before the assay, the desired transfected-CHO-745 cell line as well as the non-transfected CHO-745 cells (for preabsorption) were cultivated in culture flasks. On the day of the assay highly synchronised trophozoite-stage IEs (10% parasitaemia), were suspended in binding medium (5% haematocrit). Confluence level was controlled under light microscope (50–100% confluent monolayer). The parasite cell suspension was added to non-transfected CHO-745 cells (to allow IE pre-absorption) for 1 h at 37 °C (5% CO_2_), with orbital shaking every 15 min. Next, the pre-absorbed suspension was incubated with receptor-expressing CHO-745 cells for 1 h at 37 °C (5% CO_2_), with gentle shaking in all directions every 15 min. Thereafter, unbound IEs were removed by 5–8 washes of the binding medium. The binding of IEs to the CHO-745 cells was confirmed by examination under an inverted microscope. To enable further growth of the selected parasite populations, bound IEs were co-incubated with CHO-745 cells and cultivated following standard *Plasmodium* procedures for 24 h. On the following day, ring-stage IEs were harvested; this was followed by a depletion of the remaining CHO-745 cells using the BIOCOLL separating solution (Biochrom™). The selected IEs were cultured until 10% parasitaemia, and the entire procedure was repeated.

### RNA purification, library preparation and transcriptome analysis

Ring-stage IEs were harvested, rapidly lysed in a 20-times higher volume of pre-warmed (37 °C) TRIzol® (Invitrogen) and stored at −80 °C. RNA was isolated using the PureLink™ RNA Mini Kit (ThermoFisher Scientific), according to the manufacturer’s instructions. The quality and quantity of total RNA were assessed using the Agilent™ 2100 Bioanalyzer System with Agilent™ RNA 6000 Pico kit (Agilent Technologies). To avoid contamination with genomic DNA, the samples were treated with TURBO™ DNase (ThermoFisher Scientific) followed by magnetic bead enzymatic wash using Agencourt RNA Clean XP (Beckman Coulter). To ensure deep sequencing of mRNA, rRNA was depleted using the Ribo-Zero™ Kit (Illumina), according to the manufacturer’s instructions. The rRNA-depleted RNA samples were then purified using the RNeasy™ MinElute Cleanup Kit (Qiagen). The concentration of RNA as well as the quality of mRNA in the treated samples were assessed using the Agilent 2100™ Bioanalyzer System (Agilent™ RNA 6000 Pico kit for mRNA assay; Agilent Technologies).

The IT4 isolate libraries were constructed using the ScriptSeq™ v2 RNA-Seq Library Preparation Kit (Illumina) according to the manufacturer’s protocols. The abundance and size distribution of the fragments within the libraries were checked using the Agilent™ 2100 Bioanalyzer System (Agilent™ High Sensitivity DNA Kit; Agilent Technologies). The indexed libraries were multiplexed into one pool (4 nM concentration) of four different libraries. The pooled library was denatured and diluted according to the protocol described in the Illumina user guide. Next-generation sequencing runs were performed using Illumina Miseq with paired-end sequences. FastQC (Babraham Bioinformatics) was used for quality control, and the reads with ≥30 Phred quality score were included in the analysis.

For the 3D7 isolate, RNA was sent to BGI Genomics Co (Shenzhen, China). There, the Illumina Hiseq 4000 platform was used for sequencing, with the libraries multiplexed in two different lanes. Transcriptomes of *P. falciparum* strains IT4 and 3D7 were downloaded from PlasmoDB (http://plasmodb.org/plasmo/), and clean reads were aligned to these transcripts using Bowtie^72^. DESeq 1.18 was used for read normalisation^73^. Read pairs coherently aligned to one transcript were counted using samtools^74^. The *var* gene repertoire of *P. falciparum* strains IT4 and 3D7 is listed in Supplementary Tables S19 and S20.

### Statistical analysis

Statistical significance of the results of the binding assays was established using an unpaired *t*-test. Differential gene expression analysis was performed using a generalised linear model in DEseq 1.18^75^. Differentially expressed genes were defined as those that are significantly expressed in the receptor-enriched parasite population compared to the two controls (IT4/3D7_Ctrl, IT4/3D7_CHO-745). Benjamini-Hochberg (BH) method (basic R package version 3.3.2) was used to declare statistical significance of the analysis done for multicopy gene families^76^. This generated a BH adjusted *p-*value (padj). For this adjustment. The largest *p-*value (*p* < (i/m)Q) was considered significant and consequently all *p-*values smaller than it were also considered significant, even the ones that were bigger than their BH adjusted *p*-value^76^.

In the pie charts, the average expression of each *var* gene was displayed as a proportion of the combined *var* gene expression in each population, calculated using MS Excel 2016. Since the *var* genes IT4_*var*34 and IT4_*var*35 are most probably co-expressed in all IT4 parasite populations analysed, their expression level was deduced from the combined *var* gene expression to assess the relative expression of other *var* genes in each population.

## Electronic supplementary material

Figure S1-S7, Table S17-S20

Table S1A-G

Table S2 2A-G

Table S3

Table S4

Table S5

Table S6

Table S7

Table S8

Table S9

Table S10

Table S11

Table S12

Table S13

Table S14

Table S15

Table S16
