# Supplementary material for: Characterisation of Plasmodium falciparum populations selected on the human endothelial receptors P-selectin, E-selectin, CD9 and CD151
Source: Sci Rep. 2017 Jun 22;7:4069. doi: 10.1038/s41598-017-04241-3 (PMC5481354; doi:10.1038/s41598-017-04241-3)
Supplement: Supplementary file 1 — Figure S1-S7, Table S17-S20 [file 41598_2017_4241_MOESM1_ESM.doc]

**Characterisation of *Plasmodium falciparum* populations selected on the human endothelial receptors P-selectin, E-selectin, CD9 and CD151**

Nahla Galal Metwally^1,2#^, Ann-Kathrin Tilly^1#^, Pedro Lubiana^1^, Lisa K. Roth^1^, Michael Dörpinghaus^1^, Stephan Lorenzen^1^, Kathrin Schuldt^1^, Susanne Witt^1^, Anna Bachmann^1^, Henning Tidow^3^, Thomas Gutsmann^4^, Thorsten Burmester^5^, Thomas Roeder^6^, Egbert Tannich^1^, Iris Bruchhaus^1*^

^1^ Bernhard Nocht Institute for Tropical Medicine, Hamburg, Germany

^2^ Medical Parasitology Department, Faculty of Medicine-Suez Canal University, Ismailia, Egypt

^3^ Department of Chemistry, Institute for Biochemistry and Molecular Biology, University of Hamburg, Hamburg, Germany

^4^ Division of Biophysics, Research Center Borstel, Leibniz-Center for Medicine and Biosciences, Borstel, Germany

^5^ Institute of Zoology, Biocenter Grindel, University of Hamburg, Hamburg, Germany

^6^ Zoological Institute, Department of Molecular Physiology, Christian-Albrechts University Kiel, Kiel, Germany

*Correspndence and requests for materials should be addressed to I.B. (email:bruchhaus@bnitm.de)

^#^These authors contributed equally to this work.

Short title: P-selectin and CD9 interaction partners in *P. falciparum*

**
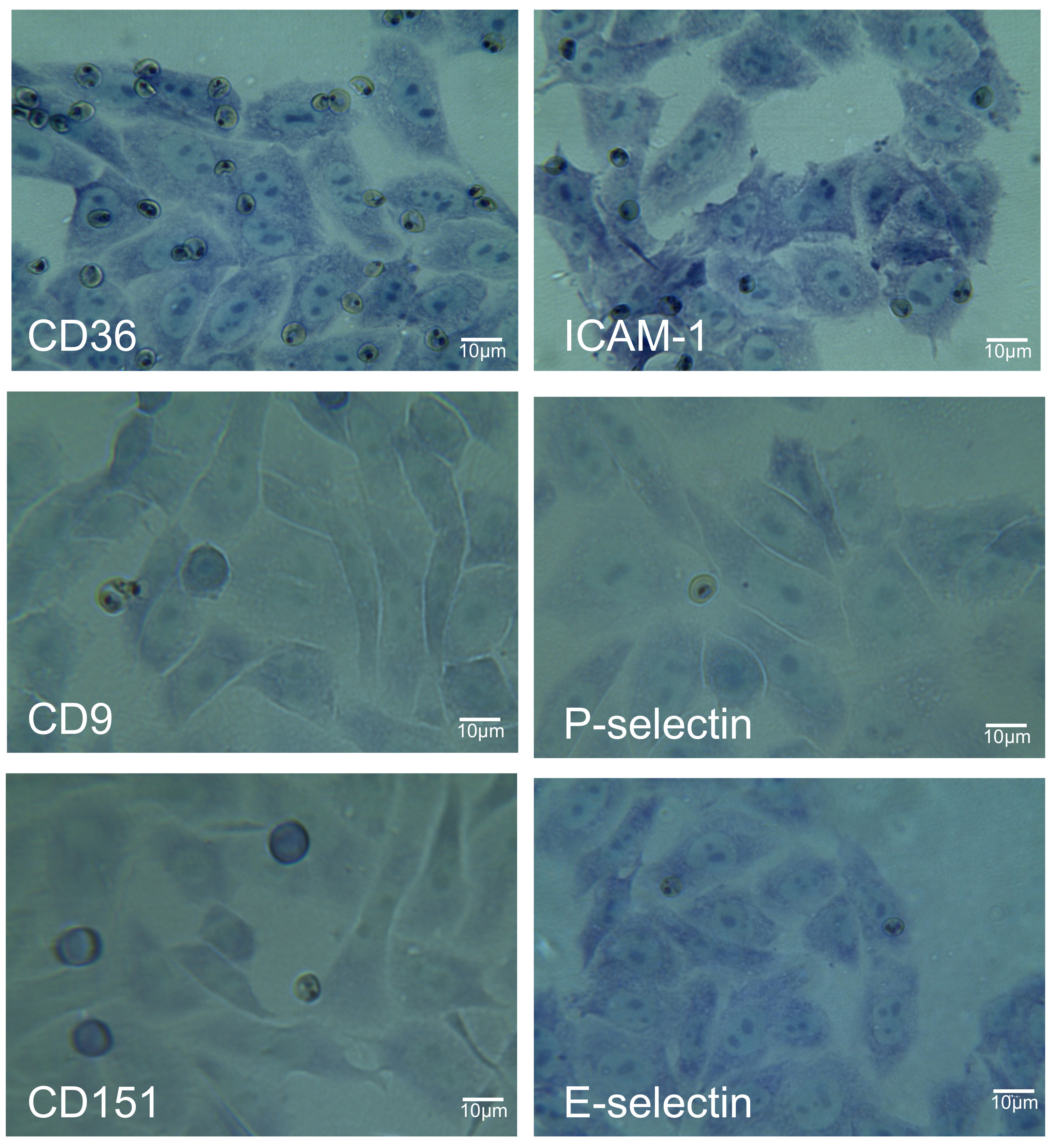
**

**Fig. S1 Cytoadhesion of erythrocytes infected with *P. falciparum* IT4 to CHO-745 cells expressing the receptor of interest (CD36, ICAM-1, CD9, P-selectin, CD151, E-selectin) on their surface.** The binding of the IEs to the various receptors was investigated before enrichment for binding to the receptor of interest. After incubation of trophozoite-stage IEs over a CHO-745 monolayer, the slides were washed, fixed with 1% glutaraldehyde and stained with Giemsa stain. Each slide was then examined under a light microscope, and the number of adherent IEs was determined by counting 300 CHO-745 cells under a light microscope. Assays were conducted three times in triplicate.

**
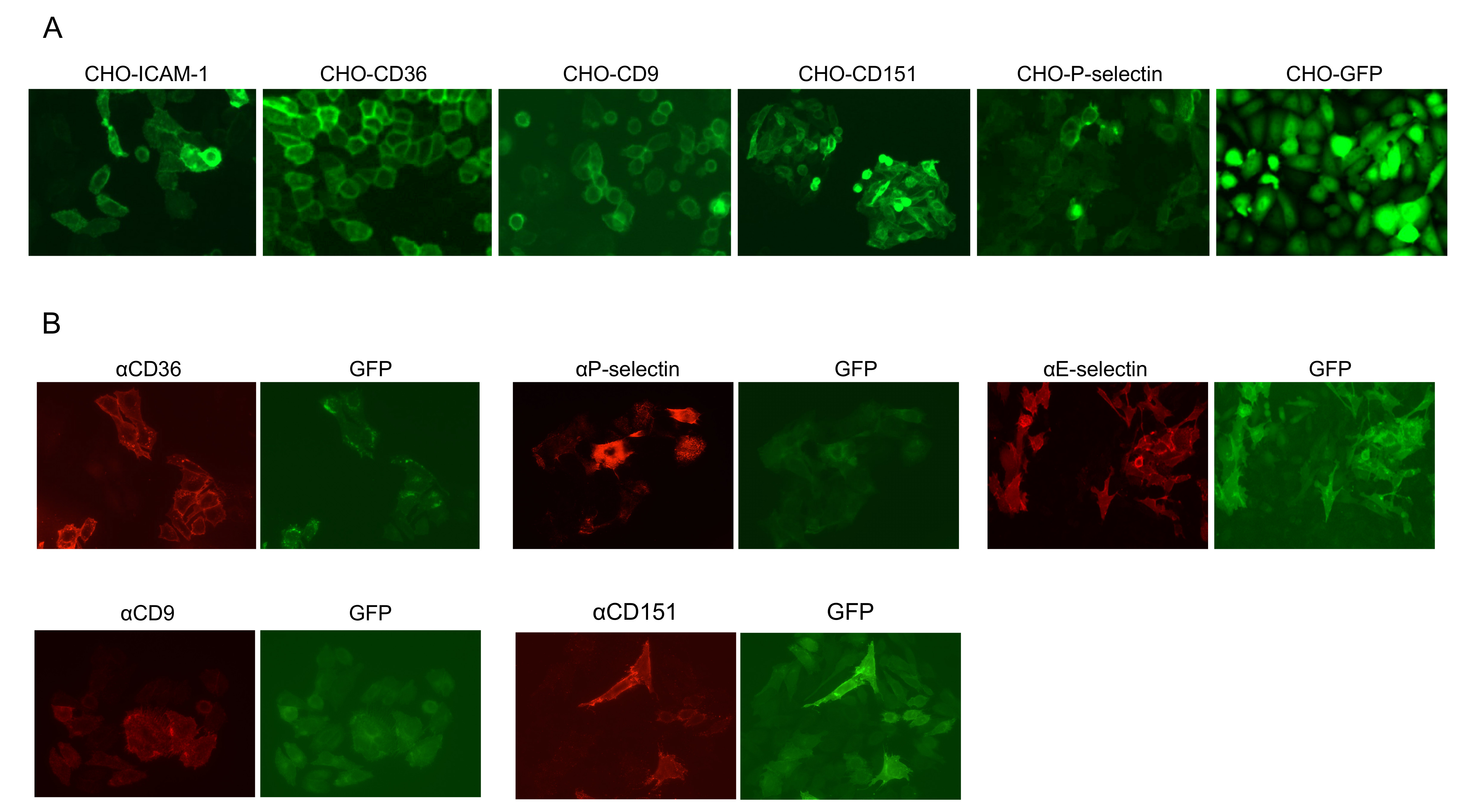
**

**Fig. S2 Immunofluorescence assays testing surface expression of human endothelial receptors on the surface of transgenic CHO-745 cells.** (A) Representative images of live CHO-745 cells expressing different human endothelial receptors (ICAM-1, CD36, CD9, CD151, P-selectin) fused to GFP on the surface. (B) Representative images of fixed CHO-745 cells expressing CD36, P-selectin, E-Selectin, CD9 and CD151 on the surface. Cells were labelled using receptor-specific antibodies and a secondary Alexa-Fluor-594 antibody.

**
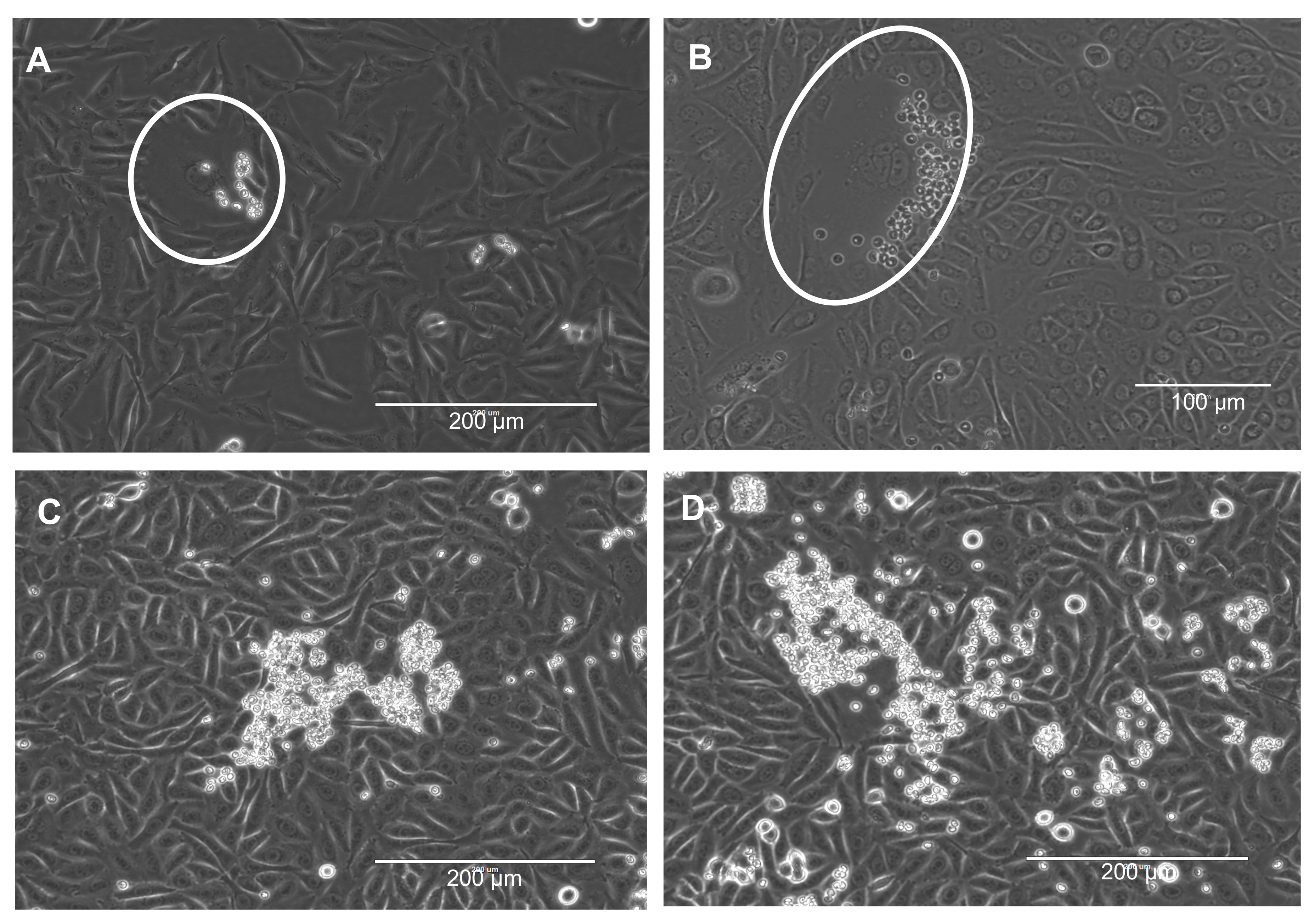
**

**Fig. S3 Enrichment of *P. falciparum* isolate IT4 during cultivation with CHO-745 WT cells** **(IT4_CHO-475).** (A, B) In the first round of selection, IEs were seen bound to a large CHO-745 cell with abnormal morphology. These senescent CHO-745 cells are marked by circles. A dramatic increase in the number of IEs bound to CHO-745 cells upon repetitive rounds of enrichment was observed in the 3^rd^ (C) and 4^th^ (D) round of enrichment (all co-incubation steps were carried out at 37 °C for 75 min, under 5% CO_2_).


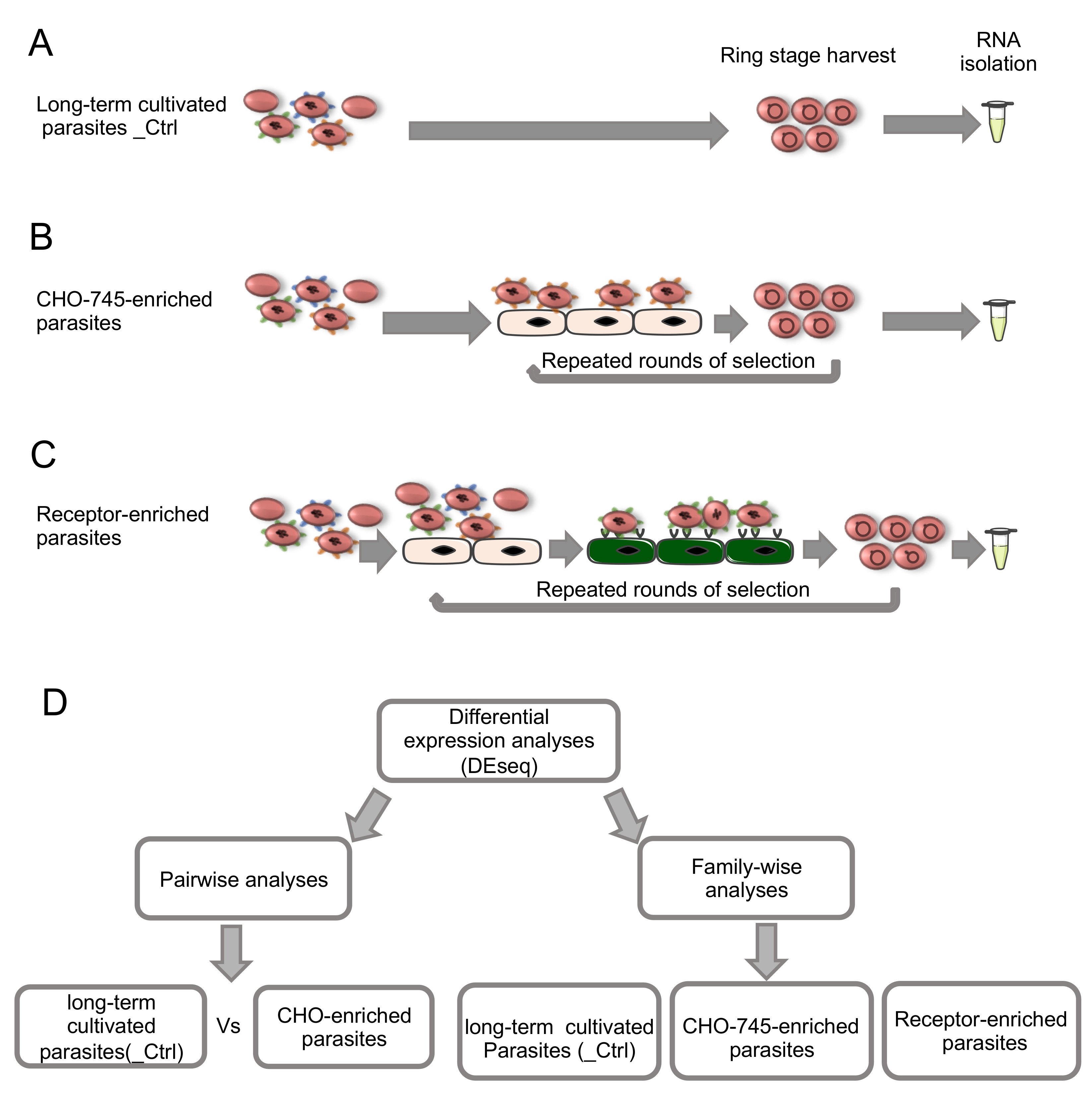


**Fig. S4 A scheme for the parasite enrichment procedure.** (A) Long-term parasite culture prepared for RNA isolation and next-generation sequencing. (B) Selection and enrichment of parasite populations cultured with CHO-745 cells. (C) Selection and enrichment of parasite populations that could bind to transgenic CHO-745 cells expressing the receptor of interest. (D) Bioinformatics scheme for differential gene expression analysis.


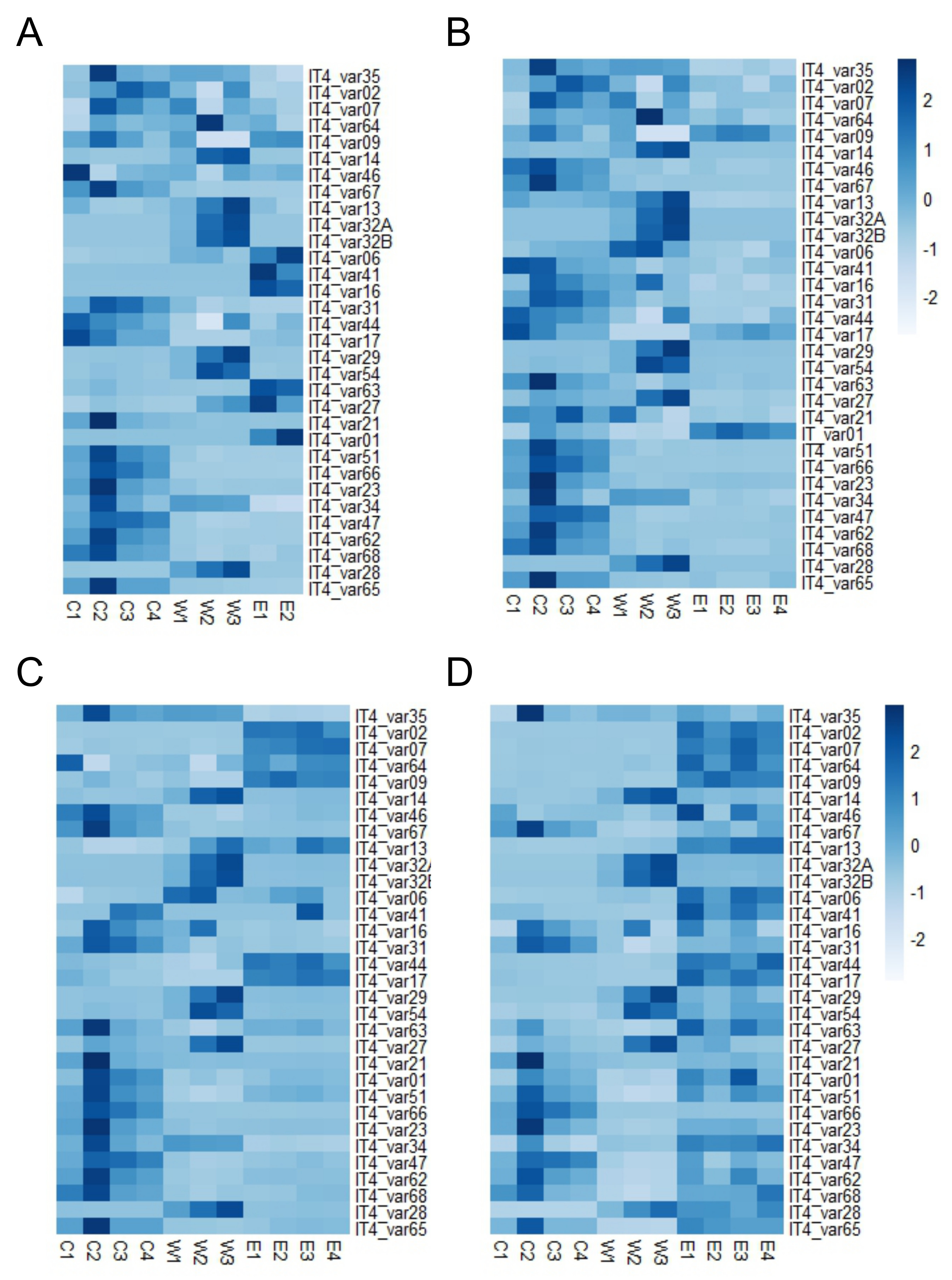


**Fig. S5 Heat maps of *var* gene expression in different parasite populations enriched for IT4 receptor-binding parasites compared with two controls populations, IT4_Ctrl and IT4_CHO.** (A) Population enriched for ICAM-1−binding parasites. (B) Population enriched for CD36-binding parasites. (C) Population enriched for P-selectin−binding parasites. (D) Population enriched for CD9-binding parasites. The heat maps were generated using R version 3.3.2 and the heatmap package. For each population, 2−4 biological replicates were analysed. C1−C4, IT4_Ctrl; W1−W3, IT4_CHO; E1−E4, receptor of interest.


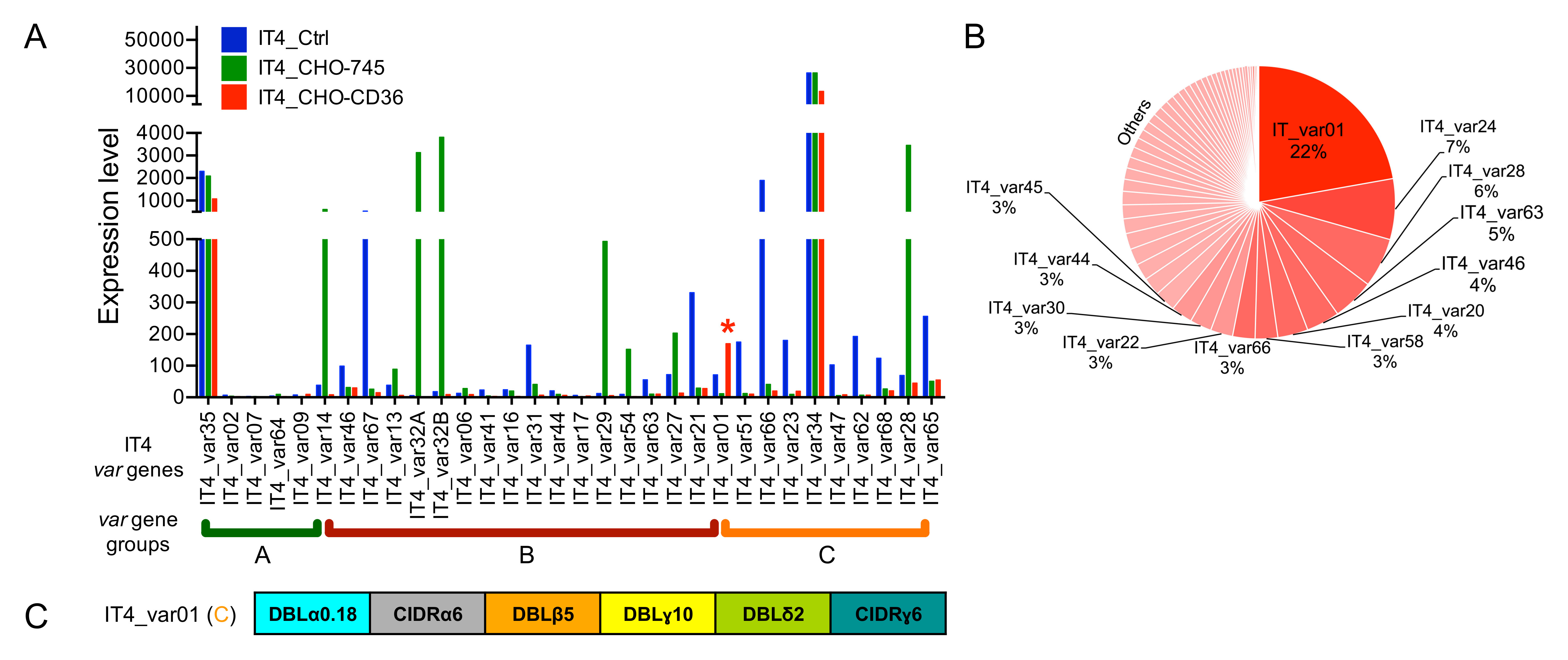


**Fig. S6.** **Enrichment of a *P. falciparum* IT4 population for CD36-binding parasites (IT4_CHO-CD36).** (A) The expression of a selected set of *var* genes in IT4_CHO-CD36 parasites in comparison with long-term cultured IT4 parasites (IT4_Crtl) and an IT4 population enriched for parasites binding to CHO-745 cells (IT4_CHO-745) (expression level is defined as average of the normalized read counts). A *var* gene whose expression was significantly increased in IT4_CHO-CD36 parasites is marked by a red star (padj < 0.05). (B) Distribution of *var* gene expression in the IT4_CHO-CD36 population. IT4_var34 and IT4_var35 genes were excluded from the analyses. (C) Schematic representation of *Pf*EMP1 protein encoded by the IT4_var01 gene with significantly higher expression in IT4_CHO-CD36 than in IT4_Crtl and IT4_CHO parasites.


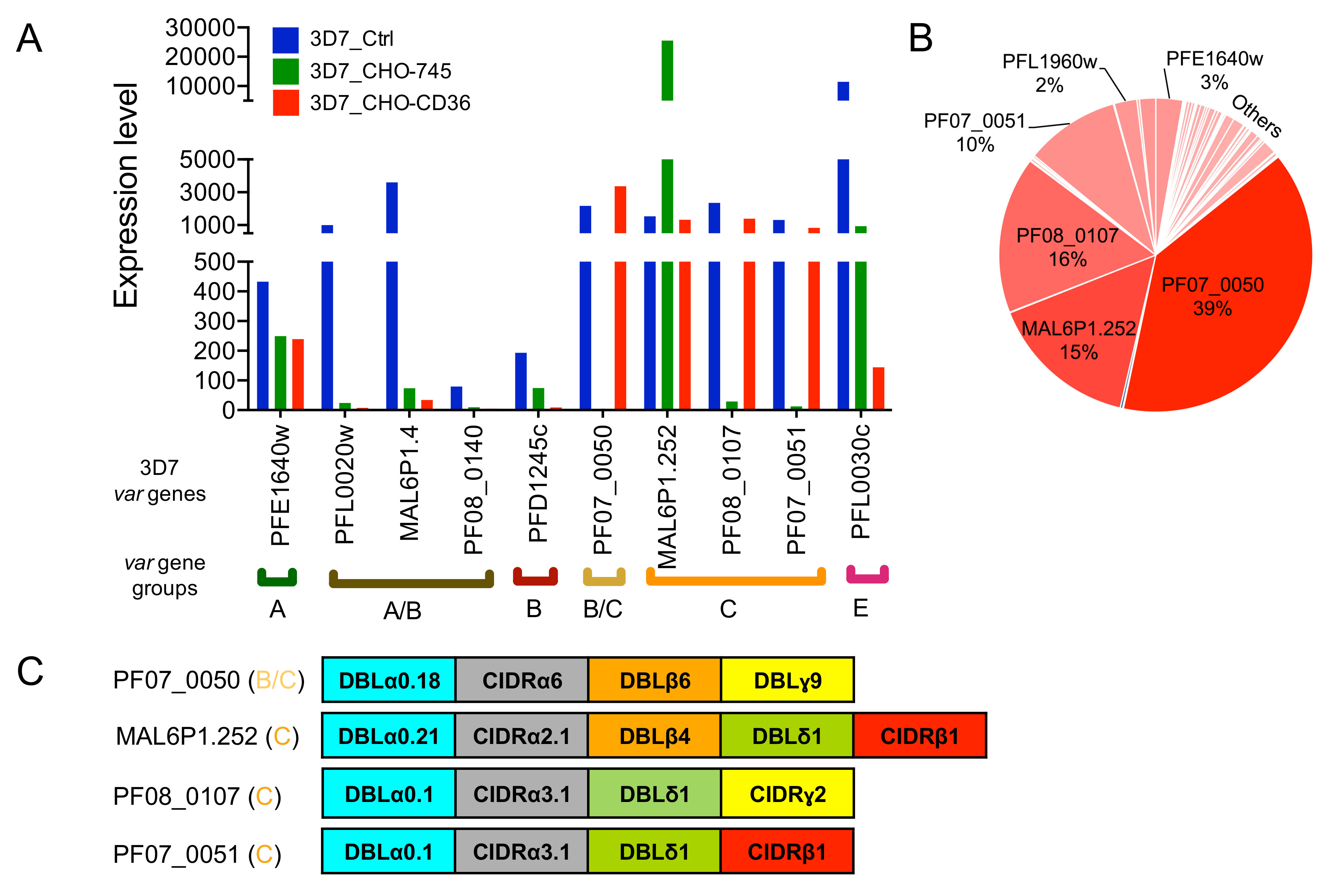


**Fig. S7.** **Enrichment of a *P. falciparum* 3D7 population for CD36-binding parasites (3D7_CHO-CD36).** (A) The expression of a selected set of *var* genes in 3D7_CHO-CD36 parasites in comparison with long-term cultured 3D7 parasites (3D7_Crtl) and a 3D7 population enriched for parasites binding to CHO-745 cells (3D7_CHO-745) (expression level is defined as average of the normalized read counts). (B) Distribution of *var* gene expression in the 3D7_CHO-CD36 population. (C) Schematic representation of *Pf*EMP1 proteins whose encoding *var* genes were dominant within the *var* gene expression profile of 3D7_CHO-CD36 parasites.

| ***rif*** | **Gene accession number** | **ICAM-1**  **-enriched** | **CD36**  **-enriched** | **P-selectin**  **-enriched** | **CD9**  **-enriched** | **E-selectin**  **-enriched** | **CD151**  **-enriched** |
| --- | --- | --- | --- | --- | --- | --- | --- |
|  | PFIT_0400300.1 |  |  |  |  |  |  |
|  | PFIT_0400300.2 |  |  |  |  |  |  |
|  | PFIT_bin04400 |  |  |  |  |  |  |
|  | PFIT_0801600 |  |  |  |  |  |  |
|  | PFIT_0800200 |  |  |  |  |  |  |
|  | PFIT_bin03400 |  |  |  |  |  |  |
|  | PFIT_bin07800 |  |  |  |  |  |  |
|  | PFIT_0616600 |  |  |  |  |  |  |
|  | PFIT_bin00500 |  |  |  |  |  |  |
|  | PFIT_0811700 |  |  |  |  |  |  |
|  | PFIT_0537100 |  |  |  |  |  |  |
|  | PFIT_1150800 |  |  |  |  |  |  |
|  | PFIT_bin04900 |  |  |  |  |  |  |
| ***stevor*** | PFIT_0901600 |  |  |  |  |  |  |
|  | PFIT_bin05800 |  |  |  |  |  |  |
| ***surf*** | PFIT_0803400 |  |  |  |  |  |  |
|  | PFIT_1301000 |  |  |  |  |  |  |
|  | PFIT_0422600 |  |  |  |  |  |  |
|  | PFIT_1301050 |  |  |  |  |  |  |
|  | PFIT_0833700 |  |  |  |  |  |  |
|  | PFIT_0400900 |  |  |  |  |  |  |

**Table S17.** **Multi-copy gene family expression in enriched IT4 *P. falciparum* populations.** Genes from the *rif*, *stevor* and *surf* gene families that showed differential expression (red) or a high expression tendency (pink) in different IT4 populations enriched for specific receptor-binding parasites in comparison with IT4_Ctrl and IT4_CHO-745, or with a consistently high expression in all IT4 populations analysed (grey).

| ***rif*** | **Gene accession number** | **ICAM-1**  **-enriched** | **CD36**  **-enriched** | **P-selectin**  **-enriched** | **CD9**  **-enriched** | **E-selectin**  **-enriched** | **CD151-enriched** |
| --- | --- | --- | --- | --- | --- | --- | --- |
|  | PF3D7_0401600.1 |  |  |  |  |  |  |
|  | PF3D7_0401600.2 |  |  |  |  |  |  |
|  | PF3D7_0400300 |  |  |  |  |  |  |
|  | PF3D7_0900200 |  |  |  |  |  |  |
|  | PF3D7_0732900 |  |  |  |  |  |  |
|  | PF3D7_0100200 |  |  |  |  |  |  |
|  | PF3D7_0800400 |  |  |  |  |  |  |
|  | PF3D7_0300200 |  |  |  |  |  |  |
| ***stevor*** | PF3D7_0102100 |  |  |  |  |  |  |
|  | PF3D7_0115400 |  |  |  |  |  |  |
| ***surf*** | PF3D7_0532800 |  |  |  |  |  |  |
|  | PF3D7_0115400 |  |  |  |  |  |  |
|  | PF3D7_1301800 |  |  |  |  |  |  |

**Table S18.** **Multi-copy gene family expression in enriched 3D7 *P. falciparum* populations.** Genes from the *rif, stevor* and *surf* gene families that showed differential expression (red) and a tendency for high expression (rose) in different 3D7 populations enriched for specific receptor-binding parasites in comparison with 3D7_Ctrl and 3D7_CHO, or with a consistently high expression in all 3D7 populations analysed (grey).

**Table S19. *Var* genes of IT4 *P. falciparum* isolate.**

|  | ***var* gene accession number** | ***var* gene ID** |
| --- | --- | --- |
|  | PFIT_1150900 | IT4_var08 |
|  | PFIT_0536800 | IT4_var35 (VAR1CSA) protein coding pseudogene |
|  | PFIT_bin02700 | IT4_var03 |
|  | PFIT_bin08900 | IT4_var02 |
|  | PFIT_1300100 | IT4_var07 |
|  | PFIT_bin10900 | IT4_var18 |
|  | PFIT_0710900 | IT4_var64 |
|  | PFIT_bin01000 | IT4_var22 |
|  | PFIT_bin06900 | IT4_var60 |
|  | PFIT_1400200 | IT4_var09 |
|  | PFIT_bin07000 | IT4_var14 |
|  | PFIT_bin11000 | IT4_var46 |
|  | PFIT_0411300 | IT4_var10 |
|  | PFIT_1240500 | IT4_var67 |
|  | PFIT_0411400 | IT4_var13 |
|  | PFIT_bin09600 | IT4_var19 |
|  | PFIT_bin00100 | IT4_var32A |
|  | PFIT_bin00900 | IT4_var32B |
|  | PFIT_bin06100 | IT4_var06 |
|  | PFIT_0900100 | IT4_var41 |
|  | PFIT_bin09100 | IT4_var16 |
|  | PFIT_bin02200 | IT4_var31 |
|  | PFIT_1151000 | IT4_var44 |
|  | PFIT_0500100 | IT4_var11 |
|  | PFIT_bin00300 | IT4_var12 |
|  | PFIT_bin07600 | IT4_var17 |
|  | PFIT_bin02100 | IT4_var29 |
|  | PFIT_bin07650 | IT4_var24 |
|  | PFIT_bin04300 | IT4_var45 |
|  | PFIT_bin04200 | IT4_var33 |
|  | PFIT_0537600 | IT4_var25 |
|  | PFIT_0835600 | IT4_var61 |
|  | PFIT_bin06200 | IT4_var54 |
|  | PFIT_1219000 | IT4_var63 |
|  | PFIT_1400100 | IT4_var26 |
|  | PFIT_0731700 | IT4_var40 |
|  | PFIT_bin10800 | IT4_var20 |
|  | PFIT_0411500 | IT4_var27 |
|  | PFIT_0411350 | IT4_var58 |
|  | PFIT_bin10500 | IT4_var21/IT4_var59 |
|  | PFIT_bin10700 | IT4_var15 |
|  | PFIT_0100100 | IT4_var36 |
|  | PFIT_1240000 | IT4_var39 |
|  | PFIT_0616500 | IT4_var01 |
|  | PFIT_bin08300 | IT4_var51 |
|  | PFIT_0710800 | IT4_var66 |
|  | PFIT_0411000 | IT4_var23 |
|  | PFIT_1240400 | IT4_var05 |
|  | PFIT_0811900 | IT4_var34 |
|  | PFIT_1241100 | IT4_var47 |
|  | PFIT_0419300 | IT4_var62 |
|  | PFIT_0710600 | IT4_var68 |
|  | PFIT_0711000 | IT4_var28 |
|  | PFIT_bin11100 | IT4_var30 |
|  | PFIT_1200200 | IT4_var04 |
|  | PFIT_0811500 | IT4_var65 |
|  | [PFIT_0800100](http://plasmodb.org/plasmo/app/record/gene/PFIT_0800100) | IT4_var13 |
|  | [PFIT_bin10300](http://plasmodb.org/plasmo/app/record/gene/PFIT_bin10300) | IT4_var41 |

**Table 20. *Var* genes of 3D7 *P. falciparum* isolate.**

|  | ***var* gene accession number** | ***var* gene ID** |
| --- | --- | --- |
|  | PF3D7_0533100 | PFE1640w (VAR1CSA) protein coding pseudogene |
|  | PF3D7_0425800 | PFD1235w |
|  | PF3D7_1150400 | PF11_0521 |
|  | PF3D7_1300300 | PF13_0003 |
|  | PF3D7_0800200 | PF08_0141 |
|  | PF3D7_1100200 | PF11_0008 |
|  | PF3D7_0400400 | PFD0020c |
|  | PF3D7_0100300 | PFA0015c |
|  | PF3D7_0600400 | MAL6P1.314 |
|  | PF3D7_0937600 | PFI1820w |
|  | PF3D7_0800300 | PF08_0140 |
|  | PF3D7_0600200 | MAL6P1.316 |
|  | PF3D7_1200400 | PFL0020w |
|  | PF3D7_0632500 | MAL6P1.4 |
|  | PF3D7_1100100 | PF11_0007 |
|  | PF3D7_0800100 | PF08_0142 |
|  | PF3D7_0500100 | PFE0005w |
|  | PF3D7_0100100 | PFA0005w |
|  | PF3D7_0115700 | PFA0765c |
|  | PF3D7_0324900 | PFC1120c |
|  | PF3D7_0400100 | PFD0005w |
|  | PF3D7_0900100 | PFI0005w |
|  | PF3D7_1373500 | PF13_0364 |
|  | PF3D7_0733000 | PF07_0139 |
|  | PF3D7_0223500 | PFB1055c |
|  | PF3D7_1041300 | PF10_0406 |
|  | PF3D7_1200100 | PFL0005w |
|  | PF3D7_0200100 | PFB0010w |
|  | PF3D7_0300100 | PFC0005w |
|  | PF3D7_1255200 | PFL2665c |
|  | PF3D7_1300100 | PF13_0001 |
|  | PF3D7_0632800 | MAL6P1.1 |
|  | PF3D7_0426000 | PFD1245c |
|  | PF3D7_0937800 | PFI1830c |
|  | PF3D7_1000100 | PF10_0001 |
|  | PF3D7_0833500 | MAL7P1.212 |
|  | PPF3D7_0700100 | MAL8P1.220 |
|  | PF3D7_1219300 | PFL0935c |
|  | PF3D7_0413100 | PFD0635c |
|  | PF3D7_1240400 | PFL1955w |
|  | PF3D7_0808700 | PF08_0106 |
|  | PF3D7_0712300 | MAL7P1.50 |
|  | PF3D7_0809100 | PF08_0103 |
|  | PF3D7_0712800 | MAL7P1.55 |
|  | PF3D7_0712400 | PF07_0050 |
|  | PF3D7_0421100 | PFD1005c |
|  | PF3D7_1240300 | PFL1950w |
|  | PF3D7_0617400 | MAL6P1.252 |
|  | PF3D7_0712900 | MAL7P1.56 |
|  | PF3D7_0808600 | PF08_0107 |
|  | PF3D7_0420700 | PFD0995c |
|  | PF3D7_0712000 | PF07_0049 |
|  | PF3D7_0412900 | PFD0630c |
|  | PF3D7_0420900 | PFD1000c |
|  | PF3D7_0421300 | PFD1015c |
|  | PF3D7_0412400 | PFD0615c |
|  | PF3D7_0712600 | PF07_0051 |
|  | PF3D7_0711700 | PF07_0048 |
|  | PF3D7_1240600 | PFL1960w |
|  | PF3D7_0412700 | PFD0625c |
|  | PF3D7_1200600 | PFL0030c (VAR2CSA) |
|  | [PF3D7_0200300](http://plasmodb.org/plasmo/app/record/gene/PF3D7_0200300) | PFB0020C |
|  | [PF3D7_0223300](http://plasmodb.org/plasmo/app/record/gene/PF3D7_0223300) | PFB1045W |
|  | [PF3D7_0600600](http://plasmodb.org/plasmo/app/record/gene/PF3D7_0600600) | MAL6P1.312 |
|  | [PF3D7_0732800](http://plasmodb.org/plasmo/app/record/gene/PF3D7_0732800) | PF07_0137 |
|  | [PF3D7_1240900](http://plasmodb.org/plasmo/app/record/gene/PF3D7_1240900) | MAL12P1.392 |
|  | [PF3D7_0200800](http://plasmodb.org/plasmo/app/record/gene/PF3D7_0200800) | PF02_0008 (protein coding pseudogene) |
|  | [PF3D7_1000900](http://plasmodb.org/plasmo/app/record/gene/PF3D7_1000900) | PF10_0011 (protein coding pseudogene) |
|  | [PF3D7_1101000](http://plasmodb.org/plasmo/app/record/gene/PF3D7_1101000) | PF11_0522 (protein coding pseudogene) |
